# Evidence of Extensive Homologous Recombination in the Core Genome of *Rickettsia*

**DOI:** 10.1155/2009/510270

**Published:** 2009-05-25

**Authors:** Jinyu Wu, Tonghai Yu, Qiyu Bao, Fangqing Zhao

**Affiliations:** ^1^Institute of Biomedical Informatics/Zhejiang Provincial Key Laboratory of Medical Genetics, Wenzhou Medical College, Wenzhou 325035, China; ^2^Center for Comparative Genomics and Bioinformatics, Department of Biochemistry and Molecular Biology, Pennsylvania State University, PA 16802, USA

## Abstract

The important role of homologous recombination has been extensively demonstrated to be fundamental for genetic variation in bacterial genomes. In contrast to extracellular or facultative intracellular bacteria, obligate intracellular bacteria are considered to be less prone to recombination, especially for their core genomes. In *Rickettsia*, only antigen-related genes were identified to have experienced homologous recombination. In this study, we employed evolutionary genomic approaches to investigate the impact of recombination on the core genome of *Rickettsia*. Phylogenetic network and phylogenetic compatibility matrix analyses are clearly consistent with the hypothesis that recombination has occurred frequently during *Rickettsia* evolution. 28% of *Rickettsia* core genes (194 out of 690) are found to present the evidence of recombination under four independent statistical methods. Further functional classification shows that these recombination events occur across all functional categories, with a significant overrepresentation in the cell wall/membrane/envelope biogenesis, which may provide a molecular basis for the parasite adaptation to host immunity. This evolutionary genomic analysis provides insight into the substantial role of recombination in the evolution of the intracellular pathogenic bacteria *Rickettsia*.

## 1. Introduction

*Rickettsia* spp. are obligate intracellular bacteria that are classified as gram-negative bacteria and belong to alpha subgroup of proteobacteria. They often live in arthropods, such as ticks, mites, louse, and fleas and can stably coexist within the host population. Some *Rickettsia* species can infect humans through bites or feces of the vectors, and then cause mild to serious diseases. It has been reported that various kinds of diseases caused by *Rickettsia* can be found worldwide. Based on phenotypic and phylogenetic evidence [1], *Rickettsia* have been classified into three different groups, including the spotted fever group (e.g., *R. africae*, *R. conorii,* and *R. massiliae*), the typhus group (e.g., *R. prowazekii* and *R. typhi*), and the unclassified group (e.g., *R. bellii*). Characterizing the evolutionary mechanism of *Rickettsia* is a key step towards understanding its adaptation to different hosts and the design of more effective drugs [2].

Homologous recombination, involved in transferring of a specific DNA fragment from one strain into the homologous region of another strain, is considered to be fundamental for generating genetic variation and to be one of the most important evolutionary mechanisms in bacterial evolution [3]. Meanwhile, it was believed that recombination is largely restricted to extracellular or facultative intracellular bacteria, such as *Escherichia coli* [4], *Mycobacterium* [5], *Helicobacter pylori* [6], and *Streptococcus* [7]. By contrast, obligate intracellular bacteria are often considered to be less prone to recombination [8, 9], and recombination cases are rarely reported. Especially, their core genomes are thought to be free of recombination. Recently, however, a completely different scenario was addressed in *Wolbachia* genome [10]. Based on four housekeeping genes (*gltA*, *dnaA*, *ftsZ*, and *groEL*) as well as other previously reported cases of *Wolbachia* recombination, Baldo et al. suggested that *Wolbachia* could be subject to widespread recombination. More recently, by investigating of multiple loci dispersed throughout the *Chlamydia trachomatis* chromosome, Gomes et al. [11] showed that recombination is widely spread in *Chlamydia trachomatis*. However, *Rickettsia*, as a group of important obligate intracellular bacteria, are often considered to have evolved with little impact of recombination [12]. Till now, only the antigens have been reported to present the evidence of recombination [13]. 

In this study, a number of evolutionary genomic methods have been employed to reveal the evidence of recombination in the evolution of *Rickettsia*. As a result, significant evidence for recombination is found among *Rickettsia* core genomes through phylogenetic network reconstruction and phylogenetic compatibility matrix analyses. We also found that 28% of the core genes may have been under recombination, which is unexpected for intracellular bacteria. Functional classification showed that recombination events occurred across all functional categories but with a significant bias in the genes involved in cell wall/membrane/envelope biogenesis. This evolutionary genomic analysis provided insight into the important role of recombination in *Rickettsia*.

## 2. Materials and Methods

12 *Rickettsia* genome sequences (*R. africae* ESF-5, *R. sibirica* 246, *R. conorii* Malish7, *R. rickettsii*, *R. massiliae* MTU5, *R. akari* Hartford, *R. felis* URRWXCal2, *R. prowazekii* Madrid E, *R. typhi* Wilmington, *R. canadensis* McKie, *R. bellii* OSU 85–389, and *R. bellii* RML369-C) were obtained from the PATRIC database (http:// patric.vbi.vt.edu/organism/genomeList.php?organismId=3&dm=Tabular). Orthologous groups of genes from different *Rickettsia* genomes were identified using OrthoMCL with the default parameters [14], which has been proven to be a very powerful tool in identifying orthologous families from multiple genomes, and it has been widely adopted in recent studies [15, 16]. Among the identified families, only the family with one-to-one orthologous relationship was considered for further analyses. The functional category of each orthologous family was obtained by BLASTing to the COG database (http://www.ncbi.nih.gov/COG/) with an E-value of 10^−5^. 

Multiple sequence alignment was performed at the amino acid level using the ClustalW program with the default settings [17]. The corresponding nucleotide sequence alignment was generated according to the protein alignment using the tranalign program implemented in the EMBOSS package [18]. To construct a phylogenetic compatibility matrix, all the orthologous gene sets were firstly concatenated into a single alignment. Then, the position of sequences in trees generated for each region was compared with that for all the other regions to produce a phylogenetic compatibility matrix using the TreeOrderScan program [19]. The phylogenetic network of the concatenated DNA sequences was constructed using the Neighbor-net method [20] implemented in the SplitsTree version 4.8 [21]. Identification of potential recombination evidence from each aligned orthologous group (at a nucleotide level) was performed using the RDP [22], MaxChi [23], Chimaera [24], and GENECONV [25] methods implemented in the RDP3 program [26]. These four programs have been indicated to be powerful in detecting recombination event and have been widely used by many studies [10, 27].

## 3. Results and Discussion

**Figure 1:**
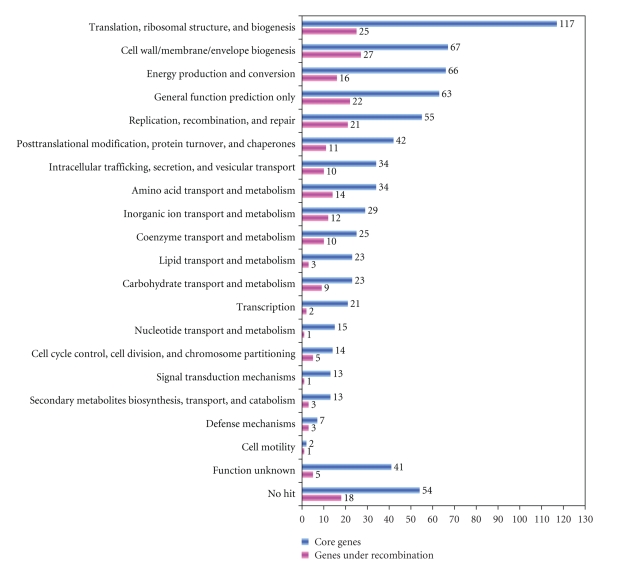
Functional classification of the *Rickettsia*'s core genes and the genes identified to be under recombination. It is notable that some proteins may be classified into multiple categories.

A core genome is a set of orthologous genes, which are shared by all members of a group of bacteria. Using the Markov Clustering algorithm [14], a total of 690 orthologous sets are found in the 12 *Rickettsia* genomes (See supplementary Table S1 in supplementary material available online at doi: 10.1155/2009/510270). This core set represents a large proportion (690/835) of the total number of ORFs in the smallest genome (*R. prowazekii* Madrid E) and nearly a half (690/1512) in the largest genome (*R. felis* URRWXCal2). In spite of the differences on the use of orthologous identification method and the number of genomes, the core set size of *Rickettsia* estimated here is quite similar to previous report [12]. Functional classification of these core sets of genes based on the COG database shows that they are present in all primary functions (Figure 1); the majority of which encode components of information-processing systems involved in translation, ribosomal structure, and biogenesis. However, there are also ~100 genes being classified into the class of “Functionally unknown” or “No hit.” 

**Figure 2:**
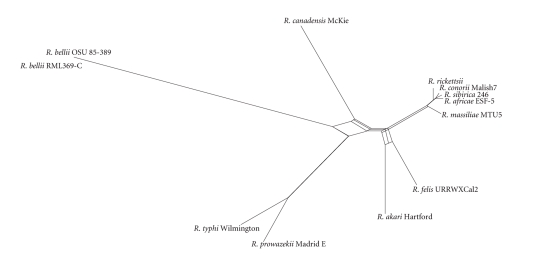
Phylogenetic network of *Rickettsia*'s core genes based on the concatenated nucleotide sequences. The phylogenetic network is constructed using the Neighbor-net method algorithm [20] and shows several parallel paths, indicating occurrence of recombination in the core genome of *Rickettsia*.

**Figure 3:**
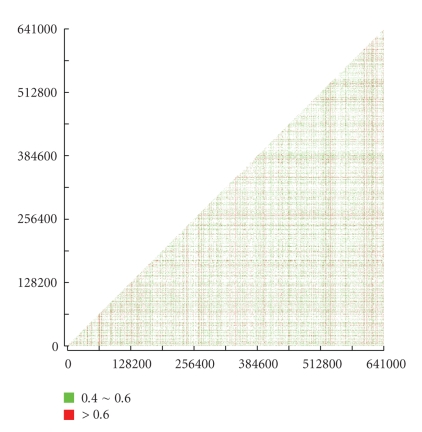
The phylogenetic compatibility matrix of *Rickettsia*'s core genes. The matrix is plotted based on the concatenated alignment of core genes with a 300-bp window and a 100-bp step. Phylogenetic trees are generated for each region using the neighbor-joining algorithm with a 70% bootstrap value. Phylogenetic violations of different topologies are calculated by comparison of the violations of the branching orders in different phylogenetic trees. Frequency is colored to indicate the number of phylogenetic violations per sequences.

A strategy for recombination detection is to construct a phylogenetic network for the target genes, which can generalize the phylogenetic tree through allowing the representation of conflicting signals or alternative phylogenetic histories [28]. To observe the phylogenetic incongruence among different gene fragments, all 690 core-set genes were concatenated into a single alignment. Using the Neighbor-net method [20], we found that multiple recombination events have occurred in the *Rickettsia* lineage (Figure 2). Meanwhile, based on the Phi test [29], it also strongly evidenced the presence of recombination in the *Rickettsia*'s core genes (*P* ≪ .00 001). Another useful strategy for detection of recombination is to generate phylogenetic trees from different parts of a gene [19]. If adjacent regions yield significantly different tree topologies, then recombination may have occurred. To visualize phylogenetic incongruence of different parts of a gene, a phylogenetic compatibility matrix was generated. As shown in Figure 3, the matrix shows a considerable number of phylogeny violations among *Rickettsia*, indicating the wide spread of recombination. Among them, about 3.4% of the phylogeny violation frequency is above 0.6 and 11.7% from 0.4 to 0.6.

To detect individual recombination event within the orthologous gene set, four recombination detection methods were used, including RDP [22], MaxChi [23], Chimaera [24], and GENECONV [25]. To ensure the reliability of our estimated results, only the recombination events that are supported by at least two independent tests were taken into account. As a result, a total of 194 core genes were found to be prone to recombination (supplementary Table S2), of which 60 gene sets were supported by four approaches and 45 gene sets could be identified by three methods. Hence, it is suggested that recombination should be prevalent than ever expected in *Rickettsia*'s core genes. Functional classification of these recombined genes clearly shows that none of the functional categories can be free from recombination (Figure 1), including the categories of translation ribosomal structure and biogenesis category. Interestingly, recombination preferentially occurs in genes involved in cell wall/membrane/envelope biogenesis. Such an elevated rate of recombination may be related with the coevolutionary arms race between *Rickettsia* and their host cells and may confer a selective advantage to survive in different niches. 

Taken together, our results strongly suggest that recombination may have played an important role in *Rickettsia* evolution. Since this study is only designed to detect the evidence of recombination events during the evolution of *Rickettsia*, the recombination breakpoints, parental and daughter sequences, as well as the possible reasons of widespread recombination in the core genome of *Rickettsia* are not considered. Combined with previous reports on *Wolbachia* [10] and *Chlamydia trachomatis* [11], we may have to update the evolutionary scenario of obligate intracellular bacteria, of which they can also be subject to extensive recombination events during evolution. The identification of the genes involved in recombination may prompt further investigation into their biological functions. 

## Supplementary Material

Supplementary Table S1 shows 690 orthologous sets found in the 12 Rickettsia genomes using the Markov Clustering algorithm. Supplementary Table S2 shows a total of 194 core genes that were found to be prone to recombination, of which 60 gene sets were supported by four approaches and 45 gene sets could be identified by three methods.
