# Supplementary material for: Evidence of Extensive Homologous Recombination in the Core Genome of Rickettsia
Source: Comp Funct Genomics. 2009 May 25;2009:510270. doi: 10.1155/2009/510270 (PMC2685993; doi:10.1155/2009/510270)
Supplement: Supplementary file 1 — Supplementary Table S1 shows 690 orthologous sets found in the 12 Rickettsia genomes using the Markov Clustering algorithm. Supplementary Table S2 shows a total of 194 core genes that were found to be prone to recombination, of which 60 gene sets were supported by four approaches and 45 gene sets could be identified by three methods. [file 510270.f1.doc]

**Table S1. The core genes shared by 12 *Rickettsia* genomes.**

| **raf** | **rak** | **rbe** | **rbo** | **rcm** | **rco** | **rfe** | **rms** | **rpr** | **rri** | **rsi** | **rty** | **COG category** |
| --- | --- | --- | --- | --- | --- | --- | --- | --- | --- | --- | --- | --- |
| ORF0001 | A1C_00005 | RBE_0001 | A1I_00005 | A1E_00005 | RC0001 | RF_0001 | RMA_0001 | RP001 | A1G_00005 | ZP_2519 | RT0001 | [S] |
| ORF0002 | A1C_00010 | RBE_0002 | A1I_00010 | A1E_00010 | RC0002 | RF_0002 | RMA_0002 | RP002 | A1G_00010 | ZP_2518 | RT0002 | [OC] |
| ORF0003 | A1C_00015 | RBE_0003 | A1I_00015 | A1E_00015 | RC0003 | RF_0003 | RMA_0003 | RP003 | A1G_00015 | ZP_2517 | RT0003 | [GM] |
| ORF0004 | A1C_00020 | RBE_0004 | A1I_00020 | A1E_00020 | RC0004 | RF_0004 | RMA_0004 | RP004 | A1G_00020 | ZP_2516 | RT0004 | [GM] |
| ORF0005 | A1C_00025 | RBE_0005 | A1I_00025 | A1E_00025 | RC0005 | RF_0005 | RMA_0005 | RP006 | A1G_00025 | ZP_2515 | RT0005 | [ER] |
| ORF0008 | A1C_00030 | RBE_1187 | A1I_01345 | A1E_00030 | RC0008 | RF_0006 | RMA_0006 | RP007 | A1G_00035 | ZP_2514 | RT0006 | [M] |
| ORF0009 | A1C_00045 | RBE_1188 | A1I_01340 | A1E_00035 | RC0009 | RF_0007 | RMA_0007 | RP008 | A1G_00040 | ZP_2513 | RT0007 | [I] |
| ORF0010 | A1C_00050 | RBE_1189 | A1I_01335 | A1E_00040 | RC0010 | RF_0008 | RMA_0008 | RP009 | A1G_00045 | ZP_2512 | RT0008 | [M] |
| ORF0013 | A1C_00305 | RBE_1286 | A1I_00440 | A1E_00050 | RC0013 | RF_0017 | RMA_0017 | RP013 | A1G_00095 | ZP_2507 | RT0012 | [R] |
| ORF0014 | A1C_00300 | RBE_0164 | A1I_07060 | A1E_00055 | RC0014 | RF_0018 | RMA_0018 | RP014 | A1G_00100 | ZP_2506 | RT0013 | [P] |
| ORF0015 | A1C_00295 | RBE_0165 | A1I_07055 | A1E_00060 | RC0015 | RF_0019 | RMA_0019 | RP015 | A1G_00105 | ZP_2505 | RT0014 | [J] |
| ORF0019 | A1C_00280 | RBE_0170 | A1I_07025 | A1E_00075 | RC0019 | RF_0022 | RMA_0020 | RP018 | A1G_00130 | ZP_2501 | RT0015 | No hit |
| ORF0022 | A1C_00265 | RBE_1293 | A1I_00400 | A1E_00085 | RC0024 | RF_0027 | RMA_0025 | RP020 | A1G_00160 | ZP_2495 | RT0108 | [C] |
| ORF0023 | A1C_00260 | RBE_1292 | A1I_00405 | A1E_00090 | RC0025 | RF_0028 | RMA_0026 | RP021 | A1G_00165 | ZP_2494 | RT0107 | [C] |
| ORF0024 | A1C_00255 | RBE_1291 | A1I_00410 | A1E_00095 | RC0026 | RF_0029 | RMA_0027 | RP022 | A1G_00170 | ZP_2493 | RT0106 | [C] |
| ORF0025 | A1C_00250 | RBE_1290 | A1I_00415 | A1E_00100 | RC0027 | RF_0030 | RMA_0028 | RP023 | A1G_00175 | ZP_2492 | RT0105 | [C] |
| ORF0026 | A1C_00245 | RBE_1289 | A1I_00420 | A1E_00105 | RC0028 | RF_0031 | RMA_0029 | RP024 | A1G_00180 | ZP_2491 | RT0104 | [S] |
| ORF0027 | A1C_00240 | RBE_1288 | A1I_00425 | A1E_00110 | RC0029 | RF_0032 | RMA_0030 | RP025 | A1G_00185 | ZP_2490 | RT0103 | [O] |
| ORF0028 | A1C_00235 | RBE_1287 | A1I_00430 | A1E_00115 | RC0030 | RF_0033 | RMA_0031 | RP026 | A1G_00190 | ZP_2489 | RT0102 | [K] |
| ORF0030 | A1C_00220 | RBE_0034 | A1I_00180 | A1E_00125 | RC0032 | RF_0035 | RMA_0033 | RP029 | A1G_00205 | ZP_2487 | RT0100 | [L] |
| ORF0035 | A1C_00195 | RBE_0031 | A1I_00165 | A1E_00130 | RC0039 | RF_0040 | RMA_0043 | RP030 | A1G_00230 | ZP_2481 | RT0099 | No hit |
| ORF0041 | A1C_00165 | RBE_0027 | A1I_00140 | A1E_00140 | RC0043 | RF_0044 | RMA_0049 | RP032 | A1G_00270 | ZP_2477 | RT0098 | [K] |
| ORF0042 | A1C_00160 | RBE_0026 | A1I_00135 | A1E_00145 | RC0044 | RF_0045 | RMA_0050 | RP033 | A1G_00275 | ZP_2476 | RT0097 | [R] |
| ORF0045 | A1C_00105 | RBE_1368 | A1I_07590 | A1E_00155 | RC0048 | RF_0156 | RMA_0056 | RP034 | A1G_00300 | ZP_2473 | RT0096 | No hit |
| ORF0052 | A1C_00065 | RBE_1372 | A1I_07620 | A1E_00165 | RC0059 | RF_0150 | RMA_0063 | RP036 | A1G_00380 | ZP_2463 | RT0094 | [O] |
| ORF0054 | A1C_00705 | RBE_1260 | A1I_00985 | A1E_00175 | RC0061 | RF_0146 | RMA_0065 | RP037 | A1G_00390 | ZP_2461 | RT0093 | [O] |
| ORF0057 | A1C_00340 | RBE_0122 | A1I_07320 | A1E_00185 | RC0064 | RF_0143 | RMA_0068 | RP039 | A1G_00405 | ZP_2458 | RT0091 | [J] |
| ORF0058 | A1C_00345 | RBE_0121 | A1I_07325 | A1E_00190 | RC0065 | RF_0142 | RMA_0069 | RP040 | A1G_00410 | ZP_2457 | RT0090 | [J] |
| ORF0059 | A1C_00350 | RBE_0120 | A1I_07330 | A1E_00195 | RC0066 | RF_0141 | RMA_0070 | RP041 | A1G_00415 | ZP_2456 | RT0089 | [J] |
| ORF0060 | A1C_00355 | RBE_1394 | A1I_07755 | A1E_00200 | RC0067 | RF_0140 | RMA_0071 | RP042 | A1G_00420 | ZP_2455 | RT0088 | [D] |
| ORF0061 | A1C_00360 | RBE_1393 | A1I_07750 | A1E_00205 | RC0068 | RF_0139 | RMA_0072 | RP043 | A1G_00425 | ZP_2454 | RT0087 | [O] |
| ORF0063 | A1C_00380 | RBE_1390 | A1I_07725 | A1E_00210 | RC0069 | RF_0136 | RMA_0075 | RP044 | A1G_00440 | ZP_2453 | RT0086 | [C] |
| ORF0065 | A1C_00390 | RBE_0015 | A1I_00070 | A1E_00215 | RC0071 | RF_0134 | RMA_0077 | RP045 | A1G_00455 | ZP_2451 | RT0085 | [S] |
| ORF0066 | A1C_00395 | RBE_0014 | A1I_00065 | A1E_00220 | RC0072 | RF_0133 | RMA_0078 | RP046 | A1G_00460 | ZP_2450 | RT0084 | [M] |
| ORF0067 | A1C_00400 | RBE_0012 | A1I_00060 | A1E_00225 | RC0073 | RF_0132 | RMA_0079 | RP047 | A1G_00470 | ZP_2449 | RT0083 | [M] |
| ORF0068 | A1C_00405 | RBE_0011 | A1I_00055 | A1E_00230 | RC0074 | RF_0131 | RMA_0080 | RP048 | A1G_00475 | ZP_2448 | RT0082 | [U] |
| ORF0069 | A1C_00410 | RBE_0010 | A1I_00050 | A1E_00235 | RC0075 | RF_0130 | RMA_0081 | RP049 | A1G_00480 | ZP_2447 | RT0081 | [I] |
| ORF0072 | A1C_00420 | RBE_1357 | A1I_00765 | A1E_00245 | RC0077 | RF_0128 | RMA_0083 | RP051 | A1G_00490 | ZP_2445 | RT0080 | No hit |
| ORF0076 | A1C_00440 | RBE_1355 | A1I_00775 | A1E_00250 | RC0081 | RF_0122 | RMA_0088 | RP053 | A1G_00515 | ZP_2440 | RT0079 | [C] |
| ORF0077 | A1C_00445 | RBE_1354 | A1I_00780 | A1E_00255 | RC0082 | RF_0121 | RMA_0089 | RP054 | A1G_00520 | ZP_2439 | RT0078 | [G] |
| ORF0078 | A1C_00450 | RBE_1353 | A1I_00785 | A1E_00260 | RC0083 | RF_0120 | RMA_0090 | RP055 | A1G_00525 | ZP_2438 | RT0077 | [F] |
| ORF0079 | A1C_00455 | RBE_1352 | A1I_00790 | A1E_00265 | RC0084 | RF_0119 | RMA_0091 | RP056 | A1G_00530 | ZP_2437 | RT0076 | [D] |
| ORF0081 | A1C_00465 | RBE_1350 | A1I_00800 | A1E_00270 | RC0086 | RF_0117 | RMA_0093 | RP058 | A1G_00540 | ZP_2435 | RT0074 | [D] |
| ORF0082 | A1C_00470 | RBE_1349 | A1I_00805 | A1E_00275 | RC0087 | RF_0116 | RMA_0094 | RP059 | A1G_00545 | ZP_2434 | RT0073 | [K] |
| ORF0083 | A1C_00475 | RBE_1348 | A1I_00810 | A1E_00280 | RC0088 | RF_0115 | RMA_0095 | RP060 | A1G_00550 | ZP_2433 | RT0072 | [R] |
| ORF0084 | A1C_00480 | RBE_1347 | A1I_00815 | A1E_00285 | RC0089 | RF_0114 | RMA_0096 | RP061 | A1G_00555 | ZP_2432 | RT0071 | No hit |
| ORF0085 | A1C_00485 | RBE_1345 | A1I_00825 | A1E_00290 | RC0090 | RF_0113 | RMA_0097 | RP062 | A1G_00560 | ZP_2431 | RT0070 | [M] |
| ORF0087 | A1C_00495 | RBE_1299 | A1I_00370 | A1E_00300 | RC0093 | RF_0111 | RMA_0101 | RP063 | A1G_00575 | ZP_2428 | RT0069 | [S] |
| ORF0088 | A1C_00500 | RBE_1301 | A1I_00360 | A1E_00305 | RC0094 | RF_0110 | RMA_0102 | RP064 | A1G_00580 | ZP_2427 | RT0068 | [F] |
| ORF0089 | A1C_00505 | RBE_1302 | A1I_00355 | A1E_00310 | RC0095 | RF_0109 | RMA_0103 | RP065 | A1G_00585 | ZP_2426 | RT0067 | [J] |
| ORF0090 | A1C_00510 | RBE_1303 | A1I_00350 | A1E_00315 | RC0096 | RF_0108 | RMA_0104 | RP066 | A1G_00590 | ZP_2425 | RT0066 | No hit |
| ORF0092 | A1C_00520 | RBE_1305 | A1I_00340 | A1E_00320 | RC0097 | RF_0107 | RMA_0105 | RP067 | A1G_00595 | ZP_2424 | RT0065 | [L] |
| ORF0094 | A1C_00535 | RBE_1298 | A1I_00375 | A1E_00325 | RC0098 | RF_0053 | RMA_0106 | RP068 | A1G_00605 | ZP_2423 | RT0064 | No hit |
| ORF0095 | A1C_00540 | RBE_1297 | A1I_00380 | A1E_00330 | RC0099 | RF_0054 | RMA_0107 | RP069 | A1G_00610 | ZP_2422 | RT0063 | [F] |
| ORF0096 | A1C_00545 | RBE_1296 | A1I_00385 | A1E_00335 | RC0100 | RF_0055 | RMA_0108 | RP070 | A1G_00615 | ZP_2421 | RT0062 | [U] |
| ORF0097 | A1C_00550 | RBE_1295 | A1I_00390 | A1E_00340 | RC0101 | RF_0056 | RMA_0109 | RP071 | A1G_00620 | ZP_2420 | RT0061 | [TK] |
| ORF0098 | A1C_00555 | RBE_1294 | A1I_00395 | A1E_00345 | RC0102 | RF_0057 | RMA_0110 | RP072 | A1G_00625 | ZP_2419 | RT0060 | [R] |
| ORF0099 | A1C_00560 | RBE_0025 | A1I_00130 | A1E_00350 | RC0103 | RF_0058 | RMA_0111 | RP073 | A1G_00630 | ZP_2418 | RT0059 | [S] |
| ORF0101 | A1C_00570 | RBE_0022 | A1I_00110 | A1E_00380 | RC0105 | RF_0060 | RMA_0113 | RP075 | A1G_00640 | ZP_2416 | RT0057 | [M] |
| ORF0102 | A1C_00575 | RBE_0021 | A1I_00105 | A1E_00385 | RC0106 | RF_0061 | RMA_0114 | RP076 | A1G_00645 | ZP_2415 | RT0056 | [GER] |
| ORF0103 | A1C_00580 | RBE_0020 | A1I_00100 | A1E_00390 | RC0107 | RF_0062 | RMA_0115 | RP077 | A1G_00650 | ZP_2414 | RT0055 | [GEPR] |
| ORF0104 | A1C_00595 | RBE_1346 | A1I_00820 | A1E_00395 | RC0109 | RF_0104 | RMA_0117 | RP079 | A1G_00665 | ZP_2413 | RT0053 | [U] |
| ORF0106 | A1C_00615 | RBE_1281 | A1I_00460 | A1E_00410 | RC0111 | RF_0068 | RMA_0120 | RP085 | A1G_00675 | ZP_2411 | RT0051 | [J] |
| ORF0107 | A1C_00620 | RBE_1282 | A1I_00455 | A1E_00415 | RC0112 | RF_0069 | RMA_0121 | RP086 | A1G_00680 | ZP_2410 | RT0050 | [J] |
| ORF0108 | A1C_00625 | RBE_1283 | A1I_00450 | A1E_00420 | RC0113 | RF_0070 | RMA_0122 | RP087 | A1G_00685 | ZP_2409 | RT0049 | [J] |
| ORF0110 | A1C_00640 | RBE_1335 | A1I_00870 | A1E_00425 | RC0118 | RF_0072 | RMA_0125 | RP089 | A1G_00700 | ZP_2406 | RT0048 | [M] |
| ORF0111 | A1C_00645 | RBE_1334 | A1I_00875 | A1E_00430 | RC0119 | RF_0073 | RMA_0126 | RP090 | A1G_00705 | ZP_2405 | RT0047 | [S] |
| ORF0112 | A1C_00650 | RBE_1333 | A1I_00880 | A1E_00435 | RC0120 | RF_0074 | RMA_0127 | RP091 | A1G_00710 | ZP_2404 | RT0046 | [E] |
| ORF0113 | A1C_00655 | RBE_1332 | A1I_00890 | A1E_00440 | RC0122 | RF_0075 | RMA_0130 | RP092 | A1G_00720 | ZP_2403 | RT0045 | [QR] |
| ORF0114 | A1C_00660 | RBE_1331 | A1I_00895 | A1E_00445 | RC0123 | RF_0076 | RMA_0131 | RP093 | A1G_00725 | ZP_2402 | RT0044 | [M] |
| ORF0115 | A1C_00665 | RBE_1330 | A1I_00900 | A1E_00450 | RC0124 | RF_0077 | RMA_0132 | RP094 | A1G_00730 | ZP_2401 | RT0043 | [Q] |
| ORF0118 | A1C_00730 | RBE_1339 | A1I_00855 | A1E_00455 | RC0128 | RF_0079 | RMA_0134 | RP095 | A1G_00750 | ZP_2398 | RT0042 | [M] |
| ORF0119 | A1C_00735 | RBE_1340 | A1I_00850 | A1E_00460 | RC0129 | RF_0080 | RMA_0135 | RP096 | A1G_00755 | ZP_2397 | RT0041 | [Q] |
| ORF0120 | A1C_00740 | RBE_1341 | A1I_00845 | A1E_00465 | RC0130 | RF_0081 | RMA_0136 | RP097 | A1G_00760 | ZP_2396 | RT0040 | [Q] |
| ORF0121 | A1C_00745 | RBE_1342 | A1I_00840 | A1E_00470 | RC0131 | RF_0082 | RMA_0137 | RP098 | A1G_00765 | ZP_2395 | RT0039 | No hit |
| ORF0124 | A1C_00750 | RBE_1279 | A1I_00470 | A1E_00475 | RC0136 | RF_0083 | RMA_0140 | RP099 | A1G_00790 | ZP_2390 | RT0038 | [J] |
| ORF0125 | A1C_00755 | RBE_1278 | A1I_00475 | A1E_00480 | RC0137 | RF_0084 | RMA_0141 | RP100 | A1G_00795 | ZP_2389 | RT0037 | [J] |
| ORF0126 | A1C_00760 | RBE_1277 | A1I_00480 | A1E_00485 | RC0138 | RF_0085 | RMA_0142 | RP102 | A1G_00800 | ZP_2388 | RT0035 | [R] |
| ORF0129 | A1C_00785 | RBE_1266 | A1I_00950 | A1E_00500 | RC0141 | RF_0088 | RMA_0145 | RP103 | A1G_00815 | ZP_2385 | RT0033 | [U] |
| ORF0130 | A1C_00790 | RBE_1265 | A1I_00955 | A1E_00505 | RC0142 | RF_0089 | RMA_0146 | RP104 | A1G_00820 | ZP_2384 | RT0032 | [U] |
| ORF0132 | A1C_00795 | RBE_1264 | A1I_00960 | A1E_00510 | RC0143 | RF_0090 | RMA_0147 | RP105 | A1G_00825 | ZP_2383 | RT0031 | [U] |
| ORF0133 | A1C_00800 | RBE_1263 | A1I_00965 | A1E_00515 | RC0144 | RF_0091 | RMA_0148 | RP106 | A1G_00830 | ZP_2382 | RT0030 | [U] |
| ORF0140 | A1C_00840 | RBE_1217 | A1I_01150 | A1E_00560 | RC0151 | RF_0099 | RMA_0158 | RP111 | A1G_00875 | ZP_2375 | RT0025 | [J] |
| ORF0141 | A1C_00845 | RBE_1218 | A1I_01155 | A1E_00565 | RC0152 | RF_0100 | RMA_0159 | RP112 | A1G_00880 | ZP_2374 | RT0024 | [J] |
| ORF0142 | A1C_00850 | RBE_1185 | A1I_01355 | A1E_00570 | RC0153 | RF_1180 | RMA_0160 | RP113 | A1G_00885 | ZP_2373 | RT0023 | [S] |
| ORF0143 | A1C_00855 | RBE_1184 | A1I_01360 | A1E_00575 | RC0154 | RF_1179 | RMA_0161 | RP114 | A1G_00890 | ZP_2372 | RT0022 | [U] |
| ORF0144 | A1C_00860 | RBE_1183 | A1I_01365 | A1E_00580 | RC0155 | RF_1178 | RMA_0162 | RP115 | A1G_00895 | ZP_2371 | RT0021 | [C] |
| ORF0145 | A1C_00865 | RBE_1182 | A1I_01370 | A1E_00585 | RC0156 | RF_1177 | RMA_0163 | RP116 | A1G_00900 | ZP_2370 | RT0020 | [U] |
| ORF0146 | A1C_00870 | RBE_1181 | A1I_01375 | A1E_00590 | RC0157 | RF_1176 | RMA_0164 | RP117 | A1G_00905 | ZP_2369 | RT0019 | [K] |
| ORF0147 | A1C_00875 | RBE_1179 | A1I_01385 | A1E_00595 | RC0158 | RF_1174 | RMA_0165 | RP118 | A1G_00910 | ZP_2368 | RT0018 | [R] |
| ORF0148 | A1C_00880 | RBE_1178 | A1I_01390 | A1E_00600 | RC0159 | RF_1173 | RMA_0166 | RP119 | A1G_00915 | ZP_2367 | RT0017 | [L] |
| ORF0151 | A1C_00895 | RBE_1174 | A1I_01410 | A1E_00610 | RC0162 | RF_1170 | RMA_0170 | RP120 | A1G_00935 | ZP_2364 | RT0109 | [M] |
| ORF0152 | A1C_00900 | RBE_1173 | A1I_01415 | A1E_00615 | RC0163 | RF_1169 | RMA_0171 | RP121 | A1G_00940 | ZP_2363 | RT0110 | [D] |
| ORF0153 | A1C_00905 | RBE_1172 | A1I_01420 | A1E_00620 | RC0164 | RF_1168 | RMA_0172 | RP122 | A1G_00945 | ZP_2362 | RT0111 | [O] |
| ORF0154 | A1C_00915 | RBE_1171 | A1I_01425 | A1E_00625 | RC0165 | RF_1167 | RMA_0173 | RP123 | A1G_00950 | ZP_2361 | RT0112 | [O] |
| ORF0155 | A1C_00920 | RBE_1170 | A1I_01430 | A1E_00630 | RC0166 | RF_1166 | RMA_0174 | RP124 | A1G_00955 | ZP_2360 | RT0113 | [O] |
| ORF0156 | A1C_00925 | RBE_1169 | A1I_01435 | A1E_00635 | RC0167 | RF_1164 | RMA_0175 | RP125 | A1G_00960 | ZP_2359 | RT0114 | [R] |
| ORF0157 | A1C_00930 | RBE_1168 | A1I_01440 | A1E_00640 | RC0168 | RF_1163 | RMA_0176 | RP126 | A1G_00965 | ZP_2358 | RT0115 | [C] |
| ORF0158 | A1C_00940 | RBE_1167 | A1I_01445 | A1E_00650 | RC0169 | RF_1161 | RMA_0177 | RP127 | A1G_00975 | ZP_2357 | RT0116 | [C] |
| ORF0159 | A1C_00955 | RBE_1164 | A1I_01460 | A1E_00655 | RC0170 | RF_1159 | RMA_0178 | RP128 | A1G_00980 | ZP_2356 | RT0117 | [C] |
| ORF0160 | A1C_00960 | RBE_1163 | A1I_01465 | A1E_00665 | RC0171 | RF_1156 | RMA_0179 | RP129 | A1G_00985 | ZP_2355 | RT0118 | [E] |
| ORF0161 | A1C_00965 | RBE_1162 | A1I_01470 | A1E_00670 | RC0172 | RF_1155 | RMA_0180 | RP130 | A1G_00990 | ZP_2354 | RT0119 | [J] |
| ORF0162 | A1C_00970 | RBE_1161 | A1I_01475 | A1E_00675 | RC0173 | RF_1154 | RMA_0181 | RP131 | A1G_00995 | ZP_2353 | RT0120 | [J] |
| ORF0163 | A1C_00975 | RBE_1160 | A1I_01480 | A1E_00680 | RC0174 | RF_1153 | RMA_0182 | RP132 | A1G_01000 | ZP_2352 | RT0121 | [J] |
| ORF0164 | A1C_00980 | RBE_1159 | A1I_01485 | A1E_00685 | RC0175 | RF_1152 | RMA_0183 | RP134 | A1G_01005 | ZP_2351 | RT0123 | [U] |
| ORF0165 | A1C_00985 | RBE_1158 | A1I_01490 | A1E_00690 | RC0176 | RF_1151 | RMA_0184 | RP135 | A1G_01010 | ZP_2350 | RT0124 | [K] |
| ORF0166 | A1C_00990 | RBE_1157 | A1I_01495 | A1E_00695 | RC0177 | RF_1150 | RMA_0185 | RP136 | A1G_01015 | ZP_2349 | RT0125 | [J] |
| ORF0167 | A1C_00995 | RBE_1156 | A1I_01500 | A1E_00700 | RC0178 | RF_1149 | RMA_0186 | RP137 | A1G_01020 | ZP_2348 | RT0126 | [J] |
| ORF0168 | A1C_01000 | RBE_1155 | A1I_01505 | A1E_00705 | RC0179 | RF_1148 | RMA_0187 | RP138 | A1G_01025 | ZP_2347 | RT0127 | [J] |
| ORF0169 | A1C_01005 | RBE_1154 | A1I_01510 | A1E_00710 | RC0180 | RF_1147 | RMA_0188 | RP139 | A1G_01030 | ZP_2346 | RT0128 | [J] |
| ORF0170 | A1C_01010 | RBE_1153 | A1I_01515 | A1E_00715 | RC0181 | RF_1146 | RMA_0189 | RP140 | A1G_01035 | ZP_2345 | RT0129 | [K] |
| ORF0171 | A1C_01015 | RBE_1152 | A1I_01520 | A1E_00720 | RC0182 | RF_1145 | RMA_0190 | RP141 | A1G_01040 | ZP_2344 | RT0130 | [K] |
| ORF0173 | A1C_01025 | RBE_0351 | A1I_06015 | A1E_00730 | RC0184 | RF_1143 | RMA_0192 | RP142 | A1G_01050 | ZP_2342 | RT0131 | [E] |
| ORF0174 | A1C_01030 | RBE_0350 | A1I_06020 | A1E_00735 | RC0185 | RF_1142 | RMA_0193 | RP143 | A1G_01055 | ZP_2341 | RT0132 | [D] |
| ORF0176 | A1C_01040 | RBE_0349 | A1I_06025 | A1E_00745 | RC0187 | RF_1138 | RMA_0195 | RP145 | A1G_01065 | ZP_2339 | RT0134 | [J] |
| ORF0178 | A1C_01050 | RBE_1139 | A1I_01610 | A1E_00750 | RC0189 | RF_1134 | RMA_0197 | RP147 | A1G_01075 | ZP_2337 | RT0136 | [R] |
| ORF0179 | A1C_01055 | RBE_1138 | A1I_01615 | A1E_00755 | RC0190 | RF_1133 | RMA_0198 | RP148 | A1G_01080 | ZP_2336 | RT0137 | [E] |
| ORF0180 | A1C_01060 | RBE_1137 | A1I_01620 | A1E_00760 | RC0191 | RF_1132 | RMA_0199 | RP149 | A1G_01085 | ZP_2335 | RT0138 | [R] |
| ORF0181 | A1C_01065 | RBE_1136 | A1I_01625 | A1E_00765 | RC0192 | RF_1131 | RMA_0200 | RP150 | A1G_01090 | ZP_2334 | RT0139 | [ET] |
| ORF0182 | A1C_01070 | RBE_1135 | A1I_01630 | A1E_00775 | RC0193 | RF_1130 | RMA_0201 | RP151 | A1G_01095 | ZP_2333 | RT0140 | [J] |
| ORF0183 | A1C_01075 | RBE_1134 | A1I_01635 | A1E_00780 | RC0194 | RF_1129 | RMA_0202 | RP152 | A1G_01100 | ZP_2332 | RT0141 | [J] |
| ORF0184 | A1C_01080 | RBE_1133 | A1I_01640 | A1E_00785 | RC0195 | RF_1128 | RMA_0203 | RP153 | A1G_01105 | ZP_2331 | RT0142 | [J] |
| ORF0186 | A1C_01090 | RBE_1132 | A1I_01645 | A1E_00790 | RC0197 | RF_1127 | RMA_0204 | RP154 | A1G_01115 | ZP_2329 | RT0143 | [J] |
| ORF0187 | A1C_01095 | RBE_1131 | A1I_01650 | A1E_00795 | RC0198 | RF_1126 | RMA_0205 | RP155 | A1G_01120 | ZP_2328 | RT0144 | [F] |
| ORF0188 | A1C_01100 | RBE_1130 | A1I_01655 | A1E_00800 | RC0199 | RF_1125 | RMA_0206 | RP156 | A1G_01125 | ZP_2327 | RT0145 | [P] |
| ORF0189 | A1C_01105 | RBE_1129 | A1I_01660 | A1E_00805 | RC0200 | RF_1124 | RMA_0207 | RP157 | A1G_01130 | ZP_2326 | RT0146 | [GEPR] |
| ORF0190 | A1C_01110 | RBE_1123 | A1I_01700 | A1E_00810 | RC0201 | RF_1123 | RMA_0208 | RP158 | A1G_01135 | ZP_2325 | RT0147 | No hit |
| ORF0193 | A1C_01125 | RBE_1117 | A1I_01730 | A1E_00815 | RC0202 | RF_1121 | RMA_0210 | RP160 | A1G_01150 | ZP_2324 | RT0150 | [M] |
| ORF0194 | A1C_01130 | RBE_1118 | A1I_01725 | A1E_00820 | RC0203 | RF_1120 | RMA_0211 | RP161 | A1G_01155 | ZP_2323 | RT0151 | [M] |
| ORF0195 | A1C_01135 | RBE_1119 | A1I_01720 | A1E_00825 | RC0204 | RF_1119 | RMA_0212 | RP162 | A1G_01160 | ZP_2322 | RT0152 | [K] |
| ORF0196 | A1C_01140 | RBE_1120 | A1I_01715 | A1E_00830 | RC0205 | RF_1118 | RMA_0213 | RP163 | A1G_01165 | ZP_2321 | RT0153 | [J] |
| ORF0197 | A1C_01145 | RBE_0640 | A1I_04345 | A1E_00840 | RC0206 | RF_1117 | RMA_0214 | RP165 | A1G_01170 | ZP_2320 | RT0157 | No hit |
| ORF0199 | A1C_01160 | RBE_1122 | A1I_01705 | A1E_00845 | RC0208 | RF_1113 | RMA_0216 | RP166 | A1G_01180 | ZP_2318 | RT0158 | [I] |
| ORF0201 | A1C_01170 | RBE_1141 | A1I_01600 | A1E_01000 | RC0210 | RF_1111 | RMA_0218 | RP168 | A1G_01190 | ZP_2316 | RT0159 | [S] |
| ORF0203 | A1C_01215 | RBE_1150 | A1I_01530 | A1E_00990 | RC0211 | RF_1109 | RMA_0220 | RP170 | A1G_01200 | ZP_2315 | RT0161 | [V] |
| ORF0204 | A1C_01220 | RBE_1149 | A1I_01535 | A1E_00985 | RC0212 | RF_1108 | RMA_0221 | RP171 | A1G_01205 | ZP_2314 | RT0162 | [L] |
| ORF0205 | A1C_01225 | RBE_1148 | A1I_01540 | A1E_00980 | RC0213 | RF_1107 | RMA_0222 | RP172 | A1G_01210 | ZP_2313 | RT0163 | [L] |
| ORF0206 | A1C_01230 | RBE_1147 | A1I_01545 | A1E_00975 | RC0214 | RF_1106 | RMA_0223 | RP173 | A1G_01215 | ZP_2312 | RT0164 | [U] |
| ORF0212 | A1C_01265 | RBE_1145 | A1I_01570 | A1E_00965 | RC0219 | RF_1100 | RMA_0231 | RP175 | A1G_01245 | ZP_2308 | RT0166 | [H] |
| ORF0215 | A1C_01280 | RBE_1095 | A1I_01850 | A1E_00950 | RC0223 | RF_1096 | RMA_0235 | RP176 | A1G_01275 | ZP_2304 | RT0167 | [C] |
| ORF0216 | A1C_01285 | RBE_1096 | A1I_01845 | A1E_00945 | RC0224 | RF_1095 | RMA_0236 | RP177 | A1G_01280 | ZP_2303 | RT0168 | [P] |
| ORF0217 | A1C_01290 | RBE_1097 | A1I_01840 | A1E_00940 | RC0225 | RF_1094 | RMA_0237 | RP178 | A1G_01285 | ZP_2302 | RT0169 | [H] |
| ORF0218 | A1C_01295 | RBE_1098 | A1I_01835 | A1E_00935 | RC0226 | RF_1093 | RMA_0238 | RP179 | A1G_01290 | ZP_2301 | RT0170 | [C] |
| ORF0219 | A1C_01300 | RBE_1099 | A1I_01830 | A1E_00930 | RC0227 | RF_1092 | RMA_0239 | RP180 | A1G_01300 | ZP_2300 | RT0171 | [C] |
| ORF0221 | A1C_01310 | RBE_1101 | A1I_01820 | A1E_00920 | RC0229 | RF_1089 | RMA_0242 | RP182 | A1G_01310 | ZP_2298 | RT0173 | [L] |
| ORF0222 | A1C_01315 | RBE_1102 | A1I_01815 | A1E_00915 | RC0230 | RF_1088 | RMA_0243 | RP183 | A1G_01315 | ZP_2297 | RT0174 | [R] |
| ORF0224 | A1C_01325 | RBE_1114 | A1I_01750 | A1E_00905 | RC0232 | RF_1085 | RMA_0245 | RP184 | A1G_01330 | ZP_2295 | RT0175 | [O] |
| ORF0225 | A1C_01330 | RBE_1113 | A1I_01755 | A1E_00900 | RC0233 | RF_1084 | RMA_0246 | RP185 | A1G_01335 | ZP_2294 | RT0176 | [O] |
| ORF0228 | A1C_01350 | RBE_1107 | A1I_01790 | A1E_00880 | RC0237 | RF_1079 | RMA_0251 | RP188 | A1G_01355 | ZP_2290 | RT0178 | No hit |
| ORF0229 | A1C_01355 | RBE_1106 | A1I_01795 | A1E_00875 | RC0238 | RF_1078 | RMA_0252 | RP189 | A1G_01360 | ZP_2289 | RT0179 | [L] |
| ORF0230 | A1C_01360 | RBE_1105 | A1I_01800 | A1E_00870 | RC0239 | RF_1077 | RMA_0253 | RP190 | A1G_01365 | ZP_2288 | RT0180 | [H] |
| ORF0232 | A1C_01375 | RBE_1103 | A1I_01810 | A1E_00860 | RC0241 | RF_1075 | RMA_0255 | RP192 | A1G_01380 | ZP_2286 | RT0181 | [U] |
| ORF0233 | A1C_01380 | RBE_1142 | A1I_01595 | A1E_00855 | RC0242 | RF_1074 | RMA_0256 | RP193 | A1G_01385 | ZP_2285 | RT0182 | No hit |
| ORF0241 | A1C_01455 | RBE_0517 | A1I_02970 | A1E_01020 | RC0258 | RF_1067 | RMA_0266 | RP196 | A1G_01480 | ZP_2275 | RT0186 | [T] |
| ORF0242 | A1C_01460 | RBE_0516 | A1I_02965 | A1E_01025 | RC0259 | RF_1066 | RMA_0267 | RP197 | A1G_01485 | ZP_2274 | RT0187 | [R] |
| ORF0243 | A1C_01465 | RBE_0515 | A1I_02960 | A1E_01030 | RC0260 | RF_1065 | RMA_0268 | RP198 | A1G_01490 | ZP_2273 | RT0188 | No hit |
| ORF0244 | A1C_01470 | RBE_0514 | A1I_02955 | A1E_01035 | RC0261 | RF_1064 | RMA_0269 | RP199 | A1G_01495 | ZP_2272 | RT0189 | [C] |
| ORF0246 | A1C_01480 | RBE_0512 | A1I_02940 | A1E_01045 | RC0263 | RF_1062 | RMA_0271 | RP201 | A1G_01505 | ZP_2270 | RT0191 | [O] |
| ORF0247 | A1C_01485 | RBE_0511 | A1I_02935 | A1E_01050 | RC0264 | RF_1061 | RMA_0272 | RP202 | A1G_01510 | ZP_2269 | RT0192 | [L] |
| ORF0248 | A1C_01490 | RBE_1094 | A1I_01855 | A1E_01055 | RC0266 | RF_1060 | RMA_0273 | RP203 | A1G_01520 | ZP_2268 | RT0193 | [L] |
| ORF0249 | A1C_01495 | RBE_1093 | A1I_01860 | A1E_01060 | RC0267 | RF_1059 | RMA_0274 | RP204 | A1G_01525 | ZP_2267 | RT0194 | [O] |
| ORF0253 | A1C_01525 | RBE_0794 | A1I_05100 | A1E_01185 | RC0273 | RF_1055 | RMA_0276 | RP206 | A1G_01555 | ZP_2264 | RT0196 | [L] |
| ORF0257 | A1C_01560 | RBE_0560 | A1I_03205 | A1E_01200 | RC0278 | RF_0324 | RMA_0279 | RP208 | A1G_01595 | ZP_2259 | RT0197 | [J] |
| ORF0258 | A1C_01565 | RBE_0561 | A1I_03210 | A1E_01205 | RC0279 | RF_0325 | RMA_0280 | RP209 | A1G_01600 | ZP_2258 | RT0198 | [J] |
| ORF0262 | A1C_01580 | RBE_0563 | A1I_03220 | A1E_01215 | RC0282 | RF_0328 | RMA_0289 | RP212 | A1G_01615 | ZP_2255 | RT0203 | [R] |
| ORF0266 | A1C_01600 | RBE_0521 | A1I_02990 | A1E_01225 | RC0286 | RF_0331 | RMA_0294 | RP214 | A1G_01640 | ZP_2250 | RT0205 | [V] |
| ORF0267 | A1C_01605 | RBE_0520 | A1I_02985 | A1E_01230 | RC0287 | RF_0332 | RMA_0295 | RP215 | A1G_01645 | ZP_2249 | RT0206 | No hit |
| ORF0268 | A1C_01610 | RBE_0519 | A1I_02980 | A1E_01235 | RC0288 | RF_0333 | RMA_0296 | RP216 | A1G_01650 | ZP_2248 | RT0207 | [C] |
| ORF0269 | A1C_01615 | RBE_0262 | A1I_06490 | A1E_01240 | RC0289 | RF_0336 | RMA_0297 | RP217 | A1G_01655 | ZP_2247 | RT0208 | [C] |
| ORF0272 | A1C_01620 | RBE_0523 | A1I_03000 | A1E_01245 | RC0292 | RF_0337 | RMA_0300 | RP218 | A1G_01670 | ZP_2244 | RT0209 | [M] |
| ORF0273 | A1C_01625 | RBE_0522 | A1I_02995 | A1E_01250 | RC0293 | RF_0338 | RMA_0301 | RP219 | A1G_01675 | ZP_2243 | RT0210 | [R] |
| ORF0274 | A1C_01630 | RBE_0534 | A1I_03060 | A1E_01255 | RC0294 | RF_0339 | RMA_0302 | RP220 | A1G_01680 | ZP_2242 | RT0211 | [F] |
| ORF0276 | A1C_01640 | RBE_0535 | A1I_03065 | A1E_01265 | RC0296 | RF_0979 | RMA_0304 | RP221 | A1G_01690 | ZP_2240 | RT0212 | [J] |
| ORF0281 | A1C_01655 | RBE_0236 | A1I_06670 | A1E_01280 | RC0304 | RF_0983 | RMA_0308 | RP222 | A1G_01725 | ZP_2233 | RT0214 | No hit |
| ORF0284 | A1C_01670 | RBE_0234 | A1I_06660 | A1E_01300 | RC0306 | RF_0985 | RMA_0312 | RP224 | A1G_01745 | ZP_2231 | RT0216 | [MU] |
| ORF0285 | A1C_01675 | RBE_0233 | A1I_06655 | A1E_01305 | RC0307 | RF_0986 | RMA_0313 | RP225 | A1G_01750 | ZP_2230 | RT0217 | [S] |
| ORF0286 | A1C_01680 | RBE_0231 | A1I_06640 | A1E_01310 | RC0308 | RF_0987 | RMA_0314 | RP226 | A1G_01760 | ZP_2229 | RT0218 | [R] |
| ORF0287 | A1C_01685 | RBE_0230 | A1I_06635 | A1E_01315 | RC0309 | RF_0988 | RMA_0315 | RP227 | A1G_01765 | ZP_2228 | RT0219 | [L] |
| ORF0288 | A1C_01690 | RBE_0229 | A1I_06630 | A1E_01320 | RC0310 | RF_0989 | RMA_0316 | RP228 | A1G_01770 | ZP_2227 | RT0220 | [M] |
| ORF0289 | A1C_01695 | RBE_0228 | A1I_06625 | A1E_01325 | RC0311 | RF_0990 | RMA_0317 | RP229 | A1G_01775 | ZP_2226 | RT0221 | [T] |
| ORF0290 | A1C_01700 | RBE_0227 | A1I_06620 | A1E_01330 | RC0312 | RF_0991 | RMA_0318 | RP230 | A1G_01780 | ZP_2225 | RT0222 | [S] |
| ORF0291 | A1C_01705 | RBE_0226 | A1I_06615 | A1E_01335 | RC0313 | RF_0992 | RMA_0319 | RP231 | A1G_01785 | ZP_2224 | RT0223 | No hit |
| ORF0292 | A1C_01710 | RBE_0225 | A1I_06610 | A1E_01340 | RC0314 | RF_0993 | RMA_0320 | RP232 | A1G_01790 | ZP_2223 | RT0224 | [S] |
| ORF0293 | A1C_01715 | RBE_0224 | A1I_06605 | A1E_01345 | RC0315 | RF_0994 | RMA_0321 | RP233 | A1G_01795 | ZP_2222 | RT0225 | [J] |
| ORF0294 | A1C_01720 | RBE_0223 | A1I_06600 | A1E_01350 | RC0316 | RF_0995 | RMA_0322 | RP234 | A1G_01800 | ZP_2221 | RT0226 | [J] |
| ORF0297 | A1C_01740 | RBE_0974 | A1I_03785 | A1E_01365 | RC0320 | RF_1051 | RMA_0327 | RP236 | A1G_01830 | ZP_2217 | RT0228 | [LR] |
| ORF0299 | A1C_01750 | RBE_0972 | A1I_03795 | A1E_01375 | RC0322 | RF_1049 | RMA_0329 | RP238 | A1G_01845 | ZP_2215 | RT0230 | [J] |
| ORF0300 | A1C_01755 | RBE_0971 | A1I_03800 | A1E_01380 | RC0323 | RF_1048 | RMA_0330 | RP239 | A1G_01850 | ZP_2214 | RT0231 | [G] |
| ORF0301 | A1C_01760 | RBE_0970 | A1I_03805 | A1E_01385 | RC0324 | RF_1047 | RMA_0331 | RP240 | A1G_01855 | ZP_2213 | RT0232 | No hit |
| ORF0302 | A1C_01765 | RBE_0969 | A1I_03810 | A1E_01390 | RC0325 | RF_1046 | RMA_0332 | RP241 | A1G_01860 | ZP_2212 | RT0233 | [I] |
| ORF0303 | A1C_01770 | RBE_0968 | A1I_03815 | A1E_01395 | RC0326 | RF_1045 | RMA_0333 | RP242 | A1G_01865 | ZP_2211 | RT0234 | [I] |
| ORF0304 | A1C_01775 | RBE_0967 | A1I_03820 | A1E_01400 | RC0327 | RF_1044 | RMA_0334 | RP243 | A1G_01870 | ZP_2210 | RT0235 | [V] |
| ORF0305 | A1C_01785 | RBE_0966 | A1I_03825 | A1E_01405 | RC0328 | RF_1040 | RMA_0336 | RP244 | A1G_01885 | ZP_2209 | RT0236 | No hit |
| ORF0306 | A1C_01790 | RBE_0965 | A1I_03830 | A1E_01410 | RC0329 | RF_1039 | RMA_0337 | RP245 | A1G_01890 | ZP_2208 | RT0237 | [T] |
| ORF0307 | A1C_01795 | RBE_0964 | A1I_03835 | A1E_01415 | RC0330 | RF_1038 | RMA_0338 | RP246 | A1G_01895 | ZP_2207 | RT0238 | [T] |
| ORF0308 | A1C_01800 | RBE_0963 | A1I_03840 | A1E_01420 | RC0331 | RF_1037 | RMA_0339 | RP247 | A1G_01900 | ZP_2206 | RT0239 | [M] |
| ORF0309 | A1C_01805 | RBE_0962 | A1I_03845 | A1E_01425 | RC0332 | RF_1036 | RMA_0340 | RP248 | A1G_01905 | ZP_2205 | RT0240 | [M] |
| ORF0310 | A1C_01810 | RBE_0961 | A1I_03850 | A1E_01430 | RC0333 | RF_1035 | RMA_0341 | RP249 | A1G_01910 | ZP_2204 | RT0241 | [M] |
| ORF0311 | A1C_01815 | RBE_0960 | A1I_03855 | A1E_01435 | RC0334 | RF_1034 | RMA_0342 | RP250 | A1G_01915 | ZP_2203 | RT0242 | [M] |
| ORF0312 | A1C_01820 | RBE_0958 | A1I_03870 | A1E_01440 | RC0335 | RF_1033 | RMA_0343 | RP251 | A1G_01920 | ZP_2202 | RT0243 | [D] |
| ORF0313 | A1C_01835 | RBE_0957 | A1I_03875 | A1E_01450 | RC0336 | RF_1031 | RMA_0344 | RP252 | A1G_01925 | ZP_2201 | RT0244 | No hit |
| ORF0314 | A1C_01840 | RBE_0956 | A1I_03880 | A1E_01455 | RC0337 | RF_1030 | RMA_0345 | RP253 | A1G_01930 | ZP_2200 | RT0245 | [C] |
| ORF0316 | A1C_01850 | RBE_0954 | A1I_03890 | A1E_01465 | RC0339 | RF_1028 | RMA_0347 | RP254 | A1G_01940 | ZP_2198 | RT0246 | [M] |
| ORF0322 | A1C_01880 | RBE_0258 | A1I_06510 | A1E_01495 | RC0346 | RF_1021 | RMA_0353 | RP260 | A1G_01980 | ZP_2196 | RT0251 | [L] |
| ORF0323 | A1C_01885 | RBE_0259 | A1I_06505 | A1E_01500 | RC0347 | RF_1020 | RMA_0354 | RP261 | A1G_01985 | ZP_2195 | RT0252 | [C] |
| ORF0324 | A1C_01890 | RBE_0260 | A1I_06500 | A1E_01505 | RC0348 | RF_1019 | RMA_0355 | RP262 | A1G_01990 | ZP_2194 | RT0253 | [C] |
| ORF0325 | A1C_01895 | RBE_0286 | A1I_06375 | A1E_01510 | RC0349 | RF_1018 | RMA_0356 | RP263 | A1G_01995 | ZP_2193 | RT0254 | [T] |
| ORF0326 | A1C_01915 | RBE_0288 | A1I_06365 | A1E_01525 | RC0352 | RF_1016 | RMA_0357 | RP264 | A1G_02015 | ZP_2190 | RT0255 | [M] |
| ORF0327 | A1C_01920 | RBE_0289 | A1I_06360 | A1E_01530 | RC0353 | RF_1015 | RMA_0358 | RP265 | A1G_02020 | ZP_2189 | RT0256 | [CE] |
| ORF0328 | A1C_01925 | RBE_0824 | A1I_05255 | A1E_01535 | RC0354 | RF_1014 | RMA_0359 | RP266 | A1G_02025 | ZP_2188 | RT0257 | [P] |
| ORF0329 | A1C_01930 | RBE_0825 | A1I_05260 | A1E_01540 | RC0355 | RF_1013 | RMA_0360 | RP267 | A1G_02030 | ZP_2187 | RT0258 | [P] |
| ORF0330 | A1C_01935 | RBE_0826 | A1I_05265 | A1E_01545 | RC0356 | RF_1012 | RMA_0361 | RP268 | A1G_02035 | ZP_2186 | RT0259 | No hit |
| ORF0331 | A1C_01940 | RBE_0827 | A1I_05270 | A1E_01550 | RC0357 | RF_1011 | RMA_0362 | RP269 | A1G_02040 | ZP_2185 | RT0260 | No hit |
| ORF0332 | A1C_01945 | RBE_0828 | A1I_05275 | A1E_01555 | RC0358 | RF_1010 | RMA_0363 | RP270 | A1G_02045 | ZP_2184 | RT0261 | [C] |
| ORF0333 | A1C_01950 | RBE_0829 | A1I_05280 | A1E_01560 | RC0359 | RF_1009 | RMA_0364 | RP271 | A1G_02050 | ZP_2183 | RT0262 | [C] |
| ORF0334 | A1C_01955 | RBE_0830 | A1I_05285 | A1E_01565 | RC0360 | RF_1007 | RMA_0366 | RP272 | A1G_02060 | ZP_2182 | RT0263 | [C] |
| ORF0337 | A1C_01965 | RBE_0837 | A1I_02735 | A1E_01570 | RC0363 | RF_1005 | RMA_0368 | RP273 | A1G_02070 | ZP_2181 | RT0264 | [O] |
| ORF0343 | A1C_01970 | RBE_0598 | A1I_03415 | A1E_01575 | RC0368 | RF_1001 | RMA_0372 | RP274 | A1G_02095 | ZP_2176 | RT0265 | [J] |
| ORF0344 | A1C_01975 | RBE_0600 | A1I_03420 | A1E_01580 | RC0369 | RF_1000 | RMA_0373 | RP275 | A1G_02100 | ZP_2175 | RT0266 | [M] |
| ORF0351 | A1C_01995 | RBE_1026 | A1I_02245 | A1E_04235 | RC0376 | RF_0453 | RMA_0387 | RP280 | A1G_02160 | ZP_2167 | RT0271 | [D] |
| ORF0352 | A1C_02000 | RBE_1023 | A1I_02255 | A1E_04230 | RC0377 | RF_0454 | RMA_0388 | RP281 | A1G_02165 | ZP_2166 | RT0272 | [E] |
| ORF0355 | A1C_02015 | RBE_0905 | A1I_02355 | A1E_04210 | RC0381 | RF_0458 | RMA_0392 | RP283 | A1G_02190 | ZP_2162 | RT0274 | [CP] |
| ORF0356 | A1C_02025 | RBE_1010 | A1I_02340 | A1E_04200 | RC0382 | RF_0460 | RMA_0393 | RP284 | A1G_02195 | ZP_2161 | RT0275 | [CP] |
| ORF0357 | A1C_02035 | RBE_1011 | A1I_02330 | A1E_04195 | RC0383 | RF_0461 | RMA_0394 | RP285 | A1G_02200 | ZP_2160 | RT0276 | [P] |
| ORF0358 | A1C_02040 | RBE_1012 | A1I_02325 | A1E_04190 | RC0384 | RF_0462 | RMA_0395 | RP286 | A1G_02205 | ZP_2159 | RT0277 | [U] |
| ORF0361 | A1C_02055 | RBE_1014 | A1I_02305 | A1E_04180 | RC0387 | RF_0465 | RMA_0398 | RP289 | A1G_02220 | ZP_2156 | RT0280 | [U] |
| ORF0362 | A1C_02060 | RBE_1015 | A1I_02300 | A1E_04175 | RC0388 | RF_0466 | RMA_0399 | RP290 | A1G_02225 | ZP_2155 | RT0281 | [U] |
| ORF0363 | A1C_02065 | RBE_1016 | A1I_02295 | A1E_04170 | RC0389 | RF_0467 | RMA_0400 | RP291 | A1G_02230 | ZP_2154 | RT0282 | [U] |
| ORF0364 | A1C_02070 | RBE_1017 | A1I_02290 | A1E_04165 | RC0390 | RF_0468 | RMA_0401 | RP292 | A1G_02235 | ZP_2153 | RT0283 | [NU] |
| ORF0365 | A1C_02075 | RBE_1018 | A1I_02285 | A1E_04160 | RC0391 | RF_0469 | RMA_0402 | RP293 | A1G_02240 | ZP_2152 | RT0284 | [U] |
| ORF0366 | A1C_02080 | RBE_1019 | A1I_02280 | A1E_04155 | RC0392 | RF_0470 | RMA_0403 | RP294 | A1G_02245 | ZP_2151 | RT0285 | [FP] |
| ORF0372 | A1C_02195 | RBE_0637 | A1I_04360 | A1E_04100 | RC0398 | RF_0480 | RMA_0407 | RP295 | A1G_02275 | ZP_2145 | RT0286 | No hit |
| ORF0373 | A1C_02200 | RBE_0638 | A1I_04355 | A1E_04095 | RC0399 | RF_0481 | RMA_0408 | RP296 | A1G_02280 | ZP_2144 | RT0287 | No hit |
| ORF0374 | A1C_02205 | RBE_0639 | A1I_04350 | A1E_04090 | RC0400 | RF_0482 | RMA_0409 | RP297 | A1G_02285 | ZP_2143 | RT0288 | [P] |
| ORF0375 | A1C_02210 | RBE_0388 | A1I_05820 | A1E_04085 | RC0401 | RF_0483 | RMA_0410 | RP298 | A1G_02290 | ZP_2142 | RT0289 | [L] |
| ORF0376 | A1C_02230 | RBE_0278 | A1I_06400 | A1E_04075 | RC0402 | RF_0486 | RMA_0411 | RP299 | A1G_02295 | ZP_2141 | RT0290 | [G] |
| ORF0379 | A1C_02260 | RBE_0275 | A1I_06415 | A1E_04060 | RC0405 | RF_0491 | RMA_0415 | RP301 | A1G_02310 | ZP_2138 | RT0292 | [F] |
| ORF0380 | A1C_02265 | RBE_0268 | A1I_06455 | A1E_04055 | RC0406 | RF_0492 | RMA_0416 | RP302 | A1G_02315 | ZP_2137 | RT0293 | [U] |
| ORF0383 | A1C_02280 | RBE_0530 | A1I_03040 | A1E_04025 | RC0409 | RF_0495 | RMA_0419 | RP305 | A1G_02330 | ZP_2134 | RT0295 | No hit |
| ORF0384 | A1C_02285 | RBE_0531 | A1I_03045 | A1E_04020 | RC0410 | RF_0496 | RMA_0420 | RP306 | A1G_02335 | ZP_2133 | RT0296 | [J] |
| ORF0385 | A1C_02290 | RBE_0532 | A1I_03050 | A1E_04015 | RC0411 | RF_0497 | RMA_0421 | RP307 | A1G_02345 | ZP_2132 | RT0297 | [E] |
| ORF0386 | A1C_02295 | RBE_0533 | A1I_03055 | A1E_04010 | RC0412 | RF_0498 | RMA_0422 | RP308 | A1G_02350 | ZP_2131 | RT0298 | [J] |
| ORF0392 | A1C_02310 | RBE_0265 | A1I_06470 | A1E_03995 | RC0422 | RF_0504 | RMA_0432 | RP309 | A1G_02400 | ZP_2128 | RT0299 | [U] |
| ORF0393 | A1C_02315 | RBE_0266 | A1I_06465 | A1E_03990 | RC0423 | RF_0505 | RMA_0433 | RP310 | A1G_02405 | ZP_2127 | RT0300 | [U] |
| ORF0397 | A1C_02340 | RBE_0822 | A1I_05240 | A1E_03970 | RC0428 | RF_0510 | RMA_0437 | RP314 | A1G_02430 | ZP_2124 | RT0304 | [M] |
| ORF0398 | A1C_02345 | RBE_0821 | A1I_05235 | A1E_03965 | RC0429 | RF_0511 | RMA_0438 | RP315 | A1G_02435 | ZP_2123 | RT0305 | [R] |
| ORF0399 | A1C_02350 | RBE_0820 | A1I_05230 | A1E_03960 | RC0430 | RF_0512 | RMA_0439 | RP316 | A1G_02440 | ZP_2122 | RT0306 | [E] |
| ORF0400 | A1C_02355 | RBE_0819 | A1I_05225 | A1E_03955 | RC0431 | RF_0513 | RMA_0440 | RP317 | A1G_02445 | ZP_2121 | RT0307 | [FGR] |
| ORF0401 | A1C_02360 | RBE_0818 | A1I_05220 | A1E_03950 | RC0432 | RF_0514 | RMA_0441 | RP318 | A1G_02450 | ZP_2120 | RT0308 | No hit |
| ORF0402 | A1C_02365 | RBE_0817 | A1I_05215 | A1E_03945 | RC0433 | RF_0515 | RMA_0442 | RP319 | A1G_02455 | ZP_2119 | RT0309 | [O] |
| ORF0403 | A1C_02370 | RBE_0816 | A1I_05210 | A1E_03940 | RC0434 | RF_0516 | RMA_0443 | RP320 | A1G_02460 | ZP_2118 | RT0310 | [O] |
| ORF0408 | A1C_02380 | RBE_0812 | A1I_05190 | A1E_03910 | RC0440 | RF_0519 | RMA_0452 | RP321 | A1G_02490 | ZP_2113 | RT0311 | [M] |
| ORF0410 | A1C_02390 | RBE_0810 | A1I_05180 | A1E_03900 | RC0442 | RF_0521 | RMA_0454 | RP322 | A1G_02500 | ZP_2111 | RT0312 | [R] |
| ORF0411 | A1C_02395 | RBE_0809 | A1I_05175 | A1E_03895 | RC0443 | RF_0522 | RMA_0455 | RP323 | A1G_02505 | ZP_2110 | RT0313 | [P] |
| ORF0416 | A1C_02435 | RBE_0711 | A1I_04650 | A1E_03870 | RC0448 | RF_0529 | RMA_0462 | RP325 | A1G_02535 | ZP_2105 | RT0316 | [J] |
| ORF0417 | A1C_02440 | RBE_0712 | A1I_04655 | A1E_03865 | RC0449 | RF_0530 | RMA_0463 | RP326 | A1G_02540 | ZP_2104 | RT0317 | [L] |
| ORF0420 | A1C_02455 | RBE_0715 | A1I_04670 | A1E_03850 | RC0451 | RF_0532 | RMA_0468 | RP327 | A1G_02555 | ZP_2102 | RT0318 | [O] |
| ORF0421 | A1C_02460 | RBE_0716 | A1I_04675 | A1E_03845 | RC0452 | RF_0533 | RMA_0469 | RP328 | A1G_02560 | ZP_2101 | RT0319 | [O] |
| ORF0422 | A1C_02465 | RBE_0808 | A1I_05170 | A1E_03835 | RC0454 | RF_0534 | RMA_0470 | RP329 | A1G_02570 | ZP_2099 | RT0320 | [R] |
| ORF0423 | A1C_02470 | RBE_0807 | A1I_05165 | A1E_03830 | RC0455 | RF_0535 | RMA_0471 | RP330 | A1G_02575 | ZP_2098 | RT0321 | [L] |
| ORF0425 | A1C_02480 | RBE_0710 | A1I_04645 | A1E_03820 | RC0456 | RF_0538 | RMA_0473 | RP332 | A1G_02590 | ZP_2097 | RT0322 | [M] |
| ORF0426 | A1C_02485 | RBE_0709 | A1I_04640 | A1E_03815 | RC0457 | RF_0539 | RMA_0474 | RP333 | A1G_02595 | ZP_2096 | RT0323 | [MG] |
| ORF0427 | A1C_02490 | RBE_0708 | A1I_04635 | A1E_03810 | RC0458 | RF_0540 | RMA_0475 | RP334 | A1G_02600 | ZP_2095 | RT0324 | [M] |
| ORF0430 | A1C_02510 | RBE_0706 | A1I_04625 | A1E_03790 | RC0461 | RF_0543 | RMA_0478 | RP339 | A1G_02615 | ZP_2092 | RT0329 | [M] |
| ORF0432 | A1C_02525 | RBE_0704 | A1I_04615 | A1E_03775 | RC0463 | RF_0545 | RMA_0480 | RP342 | A1G_02625 | ZP_2090 | RT0332 | No hit |
| ORF0433 | A1C_02530 | RBE_0703 | A1I_04610 | A1E_03770 | RC0464 | RF_0546 | RMA_0481 | RP343 | A1G_02630 | ZP_2089 | RT0333 | [R] |
| ORF0434 | A1C_02535 | RBE_0702 | A1I_04605 | A1E_03765 | RC0465 | RF_0547 | RMA_0482 | RP344 | A1G_02635 | ZP_2088 | RT0334 | [M] |
| ORF0435 | A1C_02565 | RBE_0841 | A1I_02715 | A1E_03760 | RC0468 | RF_0549 | RMA_0484 | RP345 | A1G_02650 | ZP_2086 | RT0335 | [J] |
| ORF0440 | A1C_02610 | RBE_0768 | A1I_04955 | A1E_03725 | RC0473 | RF_0555 | RMA_0489 | RP348 | A1G_02680 | ZP_2081 | RT0337 | [J] |
| ORF0442 | A1C_02625 | RBE_0766 | A1I_04940 | A1E_03715 | RC0475 | RF_0557 | RMA_0491 | RP350 | A1G_02690 | ZP_2079 | RT0339 | [L] |
| ORF0446 | A1C_02635 | RBE_0390 | A1I_05810 | A1E_03710 | RC0480 | RF_0560 | RMA_0496 | RP351 | A1G_02715 | ZP_2075 | RT0340 | [L] |
| ORF0448 | A1C_02645 | RBE_0392 | A1I_05800 | A1E_03700 | RC0481 | RF_0563 | RMA_0499 | RP353 | A1G_02735 | ZP_2074 | RT0342 | [C] |
| ORF0449 | A1C_02650 | RBE_0394 | A1I_05790 | A1E_03690 | RC0482 | RF_0564 | RMA_0501 | RP354 | A1G_02740 | ZP_2073 | RT0343 | [C] |
| ORF0450 | A1C_02665 | RBE_0396 | A1I_05780 | A1E_03680 | RC0483 | RF_0565 | RMA_0502 | RP355 | A1G_02745 | ZP_2072 | RT0344 | [C] |
| ORF0451 | A1C_02670 | RBE_0397 | A1I_05775 | A1E_03675 | RC0484 | RF_0566 | RMA_0503 | RP356 | A1G_02750 | ZP_2071 | RT0345 | [C] |
| ORF0452 | A1C_02675 | RBE_0398 | A1I_05770 | A1E_03670 | RC0485 | RF_0567 | RMA_0504 | RP357 | A1G_02755 | ZP_2070 | RT0346 | [C] |
| ORF0453 | A1C_02680 | RBE_0399 | A1I_05760 | A1E_03665 | RC0486 | RF_0568 | RMA_0505 | RP358 | A1G_02760 | ZP_2069 | RT0347 | [M] |
| ORF0454 | A1C_02685 | RBE_0400 | A1I_05755 | A1E_03660 | RC0487 | RF_0569 | RMA_0506 | RP359 | A1G_02765 | ZP_2068 | RT0348 | [S] |
| ORF0459 | A1C_02705 | RBE_1002 | A1I_03635 | A1E_03655 | RC0491 | RF_0571 | RMA_0509 | RP361 | A1G_02785 | ZP_2064 | RT0350 | [L] |
| ORF0460 | A1C_02710 | RBE_1001 | A1I_03640 | A1E_03650 | RC0492 | RF_0572 | RMA_0510 | RP363 | A1G_02790 | ZP_2063 | RT0351 | No hit |
| ORF0461 | A1C_02715 | RBE_1000 | A1I_03645 | A1E_03645 | RC0493 | RF_0574 | RMA_0511 | RP364 | A1G_02795 | ZP_2062 | RT0352 | No hit |
| ORF0462 | A1C_02720 | RBE_1255 | A1I_01015 | A1E_03635 | RC0494 | RF_0575 | RMA_0512 | RP365 | A1G_02800 | ZP_2061 | RT0353 | [I] |
| ORF0463 | A1C_02725 | RBE_1256 | A1I_01010 | A1E_03630 | RC0495 | RF_0576 | RMA_0513 | RP366 | A1G_02805 | ZP_2060 | RT0354 | [M] |
| ORF0472 | A1C_02755 | RBE_0871 | A1I_02545 | A1E_03595 | RC0505 | RF_0582 | RMA_0525 | RP371 | A1G_02865 | ZP_2053 | RT0359 | [J] |
| ORF0473 | A1C_02760 | RBE_0870 | A1I_02550 | A1E_03590 | RC0506 | RF_0583 | RMA_0526 | RP372 | A1G_02870 | ZP_2052 | RT0360 | [R] |
| ORF0474 | A1C_02765 | RBE_0869 | A1I_02555 | A1E_03585 | RC0507 | RF_0584 | RMA_0527 | RP373 | A1G_02875 | ZP_2051 | RT0361 | [C] |
| ORF0477 | A1C_02795 | RBE_0940 | A1I_03965 | A1E_03575 | RC0512 | RF_0586 | RMA_0532 | RP375 | A1G_02900 | ZP_2048 | RT0363 | [GEPR] |
| ORF0481 | A1C_02825 | RBE_0644 | A1I_04320 | A1E_03540 | RC0520 | RF_0590 | RMA_0536 | RP376 | A1G_02950 | ZP_2041 | RT0365 | [C] |
| ORF0483 | A1C_02830 | RBE_0990 | A1I_03705 | A1E_03535 | RC0522 | RF_0592 | RMA_0540 | RP377 | A1G_02965 | ZP_2040 | RT0366 | [C] |
| ORF0485 | A1C_02855 | RBE_0988 | A1I_03715 | A1E_03515 | RC0524 | RF_0594 | RMA_0542 | RP379 | A1G_02975 | ZP_2038 | RT0368 | [M] |
| ORF0489 | A1C_02870 | RBE_0803 | A1I_05145 | A1E_03500 | RC0527 | RF_0599 | RMA_0545 | RP383 | A1G_02990 | ZP_2035 | RT0371 | [H] |
| ORF0490 | A1C_02875 | RBE_0804 | A1I_05150 | A1E_03495 | RC0528 | RF_0600 | RMA_0546 | RP384 | A1G_02995 | ZP_2034 | RT0372 | [J] |
| ORF0493 | A1C_02895 | RBE_0937 | A1I_03980 | A1E_03475 | RC0531 | RF_0604 | RMA_0549 | RP385 | A1G_03010 | ZP_2031 | RT0374 | [L] |
| ORF0495 | A1C_02910 | RBE_0934 | A1I_03990 | A1E_03460 | RC0533 | RF_0606 | RMA_0550 | RP386 | A1G_03025 | ZP_2029 | RT0375 | [L] |
| ORF0496 | A1C_02915 | RBE_0933 | A1I_03995 | A1E_03455 | RC0534 | RF_0607 | RMA_0551 | RP387 | A1G_03030 | ZP_2028 | RT0376 | [V] |
| ORF0498 | A1C_02925 | RBE_0931 | A1I_04005 | A1E_03445 | RC0536 | RF_0609 | RMA_0553 | RP389 | A1G_03040 | ZP_2026 | RT0377 | [M] |
| ORF0499 | A1C_02930 | RBE_0322 | A1I_06185 | A1E_03440 | RC0537 | RF_0611 | RMA_0554 | RP390 | A1G_03045 | ZP_2025 | RT0378 | [M] |
| ORF0500 | A1C_02935 | RBE_0323 | A1I_06180 | A1E_03435 | RC0538 | RF_0612 | RMA_0555 | RP391 | A1G_03050 | ZP_2024 | RT0379 | [R] |
| ORF0501 | A1C_02940 | RBE_0324 | A1I_06175 | A1E_03430 | RC0539 | RF_0613 | RMA_0556 | RP392 | A1G_03060 | ZP_2023 | RT0380 | [D] |
| ORF0502 | A1C_02945 | RBE_0325 | A1I_06170 | A1E_03425 | RC0540 | RF_0614 | RMA_0557 | RP393 | A1G_03065 | ZP_2022 | RT0381 | [P] |
| ORF0503 | A1C_02950 | RBE_0326 | A1I_06165 | A1E_03420 | RC0541 | RF_0615 | RMA_0558 | RP394 | A1G_03070 | ZP_2021 | RT0382 | No hit |
| ORF0504 | A1C_02960 | RBE_0327 | A1I_06155 | A1E_03410 | RC0542 | RF_0616 | RMA_0559 | RP395 | A1G_03075 | ZP_2020 | RT0383 | No hit |
| ORF0505 | A1C_02965 | RBE_0329 | A1I_06140 | A1E_03405 | RC0543 | RF_0617 | RMA_0560 | RP396 | A1G_03080 | ZP_2019 | RT0384 | [Q] |
| ORF0506 | A1C_02970 | RBE_0330 | A1I_06135 | A1E_03400 | RC0544 | RF_0618 | RMA_0561 | RP397 | A1G_03085 | ZP_2018 | RT0385 | [OC] |
| ORF0507 | A1C_02975 | RBE_0331 | A1I_06130 | A1E_03395 | RC0545 | RF_0619 | RMA_0562 | RP398 | A1G_03090 | ZP_2017 | RT0386 | [OU] |
| ORF0508 | A1C_02980 | RBE_0332 | A1I_06125 | A1E_03390 | RC0546 | RF_0620 | RMA_0563 | RP399 | A1G_03095 | ZP_2016 | RT0387 | [F] |
| ORF0509 | A1C_02985 | RBE_0579 | A1I_03310 | A1E_03385 | RC0547 | RF_0621 | RMA_0564 | RP400 | A1G_03100 | ZP_2015 | RT0388 | [M] |
| ORF0511 | A1C_02995 | RBE_0581 | A1I_03325 | A1E_03375 | RC0549 | RF_0623 | RMA_0566 | RP402 | A1G_03110 | ZP_2013 | RT0390 | [V] |
| ORF0512 | A1C_03000 | RBE_0584 | A1I_03340 | A1E_03370 | RC0550 | RF_0624 | RMA_0567 | RP403 | A1G_03115 | ZP_2012 | RT0391 | [L] |
| ORF0514 | A1C_03010 | RBE_0894 | A1I_02420 | A1E_03365 | RC0552 | RF_0626 | RMA_0569 | RP404 | A1G_03125 | ZP_2010 | RT0392 | [R] |
| ORF0519 | A1C_03035 | RBE_0887 | A1I_02460 | A1E_03345 | RC0557 | RF_0631 | RMA_0574 | RP407 | A1G_03145 | ZP_2005 | RT0393 | [M] |
| ORF0520 | A1C_03040 | RBE_0886 | A1I_02465 | A1E_03340 | RC0558 | RF_0632 | RMA_0575 | RP408 | A1G_03150 | ZP_2004 | RT0394 | [MU] |
| ORF0521 | A1C_03045 | RBE_0885 | A1I_02470 | A1E_03335 | RC0559 | RF_0633 | RMA_0576 | RP409 | A1G_03155 | ZP_2003 | RT0395 | No hit |
| ORF0522 | A1C_03050 | RBE_0881 | A1I_02485 | A1E_03330 | RC0560 | RF_0634 | RMA_0577 | RP410 | A1G_03160 | ZP_2002 | RT0396 | [M] |
| ORF0523 | A1C_03055 | RBE_0879 | A1I_02495 | A1E_03315 | RC0561 | RF_0635 | RMA_0578 | RP411 | A1G_03165 | ZP_2001 | RT0397 | [D] |
| ORF0524 | A1C_03060 | RBE_0878 | A1I_02500 | A1E_03310 | RC0562 | RF_0636 | RMA_0579 | RP412 | A1G_03170 | ZP_2000 | RT0398 | [M] |
| ORF0525 | A1C_03065 | RBE_0877 | A1I_02505 | A1E_03305 | RC0563 | RF_0637 | RMA_0580 | RP413 | A1G_03175 | ZP_1999 | RT0399 | No hit |
| ORF0536 | A1C_03120 | RBE_0651 | A1I_04285 | A1E_03295 | RC0575 | RF_0644 | RMA_0592 | RP414 | A1G_03250 | ZP_1989 | RT0400 | [M] |
| ORF0540 | A1C_03130 | RBE_0652 | A1I_04280 | A1E_03280 | RC0579 | RF_0645 | RMA_0596 | RP415 | A1G_03270 | ZP_1985 | RT0401 | [E] |
| ORF0541 | A1C_03135 | RBE_0653 | A1I_04275 | A1E_03275 | RC0580 | RF_0646 | RMA_0597 | RP416 | A1G_03275 | ZP_1984 | RT0402 | [J] |
| ORF0542 | A1C_03140 | RBE_0654 | A1I_04270 | A1E_03270 | RC0581 | RF_0647 | RMA_0598 | RP417 | A1G_03280 | ZP_1983 | RT0403 | [J] |
| ORF0543 | A1C_03145 | RBE_0655 | A1I_04265 | A1E_03265 | RC0582 | RF_0648 | RMA_0599 | RP418 | A1G_03285 | ZP_1982 | RT0404 | [J] |
| ORF0544 | A1C_03150 | RBE_0656 | A1I_04260 | A1E_03260 | RC0583 | RF_0649 | RMA_0600 | RP419 | A1G_03290 | ZP_1981 | RT0405 | [L] |
| ORF0545 | A1C_03155 | RBE_0310 | A1I_06250 | A1E_03255 | RC0584 | RF_0650 | RMA_0601 | RP420 | A1G_03295 | ZP_1980 | RT0406 | No hit |
| ORF0547 | A1C_03165 | RBE_0748 | A1I_04845 | A1E_03245 | RC0587 | RF_0653 | RMA_0603 | RP422 | A1G_03310 | ZP_1977 | RT0408 | [J] |
| ORF0548 | A1C_03170 | RBE_0747 | A1I_04840 | A1E_03240 | RC0588 | RF_0654 | RMA_0604 | RP423 | A1G_03315 | ZP_1976 | RT0409 | [D] |
| ORF0549 | A1C_03175 | RBE_0746 | A1I_04835 | A1E_03235 | RC0589 | RF_0655 | RMA_0605 | RP424 | A1G_03320 | ZP_1975 | RT0410 | [I] |
| ORF0550 | A1C_03180 | RBE_0745 | A1I_04830 | A1E_03230 | RC0590 | RF_0656 | RMA_0606 | RP425 | A1G_03325 | ZP_1974 | RT0411 | [I] |
| ORF0551 | A1C_03185 | RBE_0744 | A1I_04825 | A1E_03225 | RC0592 | RF_0657 | RMA_0607 | RP426 | A1G_03330 | ZP_1973 | RT0412 | [T] |
| ORF0552 | A1C_03190 | RBE_0742 | A1I_04820 | A1E_03220 | RC0593 | RF_0658 | RMA_0608 | RP427 | A1G_03335 | ZP_1972 | RT0413 | [TK] |
| ORF0553 | A1C_03195 | RBE_0741 | A1I_04815 | A1E_03215 | RC0594 | RF_0659 | RMA_0609 | RP428 | A1G_03340 | ZP_1971 | RT0414 | [EH] |
| ORF0554 | A1C_03200 | RBE_0740 | A1I_04805 | A1E_03210 | RC0595 | RF_0660 | RMA_0610 | RP429 | A1G_03345 | ZP_1970 | RT0415 | [EM] |
| ORF0555 | A1C_03205 | RBE_0739 | A1I_04800 | A1E_03205 | RC0596 | RF_0661 | RMA_0611 | RP430 | A1G_03350 | ZP_1969 | RT0416 | [O] |
| ORF0556 | A1C_03210 | RBE_0738 | A1I_04795 | A1E_03200 | RC0597 | RF_0662 | RMA_0612 | RP431 | A1G_03355 | ZP_1968 | RT0417 | [O] |
| ORF0557 | A1C_03215 | RBE_1227 | A1I_01110 | A1E_03185 | RC0598 | RF_0664 | RMA_0613 | RP432 | A1G_03360 | ZP_1967 | RT0419 | [C] |
| ORF0558 | A1C_03220 | RBE_1228 | A1I_01105 | A1E_03180 | RC0599 | RF_0665 | RMA_0614 | RP433 | A1G_03365 | ZP_1966 | RT0420 | [C] |
| ORF0564 | A1C_03235 | RBE_1234 | A1I_01080 | A1E_03170 | RC0604 | RF_0668 | RMA_0619 | RP434 | A1G_03400 | ZP_1961 | RT0421 | [S] |
| ORF0567 | A1C_03240 | RBE_1235 | A1I_01075 | A1E_03165 | RC0606 | RF_0669 | RMA_0620 | RP435 | A1G_03415 | ZP_1959 | RT0422 | [J] |
| ORF0568 | A1C_03245 | RBE_1236 | A1I_01070 | A1E_03160 | RC0607 | RF_0670 | RMA_0621 | RP436 | A1G_03420 | ZP_1958 | RT0423 | [S] |
| ORF0569 | A1C_03250 | RBE_1237 | A1I_01065 | A1E_03155 | RC0608 | RF_0671 | RMA_0622 | RP437 | A1G_03425 | ZP_1957 | RT0424 | [S] |
| ORF0570 | A1C_03255 | RBE_1238 | A1I_01060 | A1E_03150 | RC0609 | RF_0672 | RMA_0623 | RP438 | A1G_03430 | ZP_1956 | RT0425 | [L] |
| ORF0571 | A1C_03295 | RBE_1251 | A1I_01030 | A1E_03130 | RC0612 | RF_0675 | RMA_0626 | RP440 | A1G_03460 | ZP_1952 | RT0427 | [G] |
| ORF0572 | A1C_03300 | RBE_1252 | A1I_01025 | A1E_03125 | RC0613 | RF_0676 | RMA_0627 | RP441 | A1G_03465 | ZP_1951 | RT0428 | [R] |
| ORF0574 | A1C_03325 | RBE_0443 | A1I_05490 | A1E_03110 | RC0616 | RF_0679 | RMA_0629 | RP443 | A1G_03475 | ZP_1949 | RT0430 | [R] |
| ORF0575 | A1C_03330 | RBE_0444 | A1I_05485 | A1E_03105 | RC0617 | RF_0680 | RMA_0630 | RP444 | A1G_03480 | ZP_1948 | RT0431 | [R] |
| ORF0576 | A1C_03335 | RBE_0445 | A1I_05480 | A1E_03100 | RC0618 | RF_0681 | RMA_0631 | RP445 | A1G_03485 | ZP_1947 | RT0432 | [O] |
| ORF0579 | A1C_03380 | RBE_0687 | A1I_04515 | A1E_03040 | RC0623 | RF_0686 | RMA_0635 | RP446 | A1G_03505 | ZP_1942 | RT0433 | [M] |
| ORF0580 | A1C_03385 | RBE_0685 | A1I_04505 | A1E_03035 | RC0624 | RF_0687 | RMA_0636 | RP447 | A1G_03510 | ZP_1941 | RT0434 | [L] |
| ORF0582 | A1C_03405 | RBE_0684 | A1I_04500 | A1E_03025 | RC0627 | RF_0689 | RMA_0637 | RP448 | A1G_03530 | ZP_1939 | RT0435 | No hit |
| ORF0583 | A1C_03410 | RBE_0798 | A1I_05120 | A1E_03020 | RC0628 | RF_0691 | RMA_0638 | RP449 | A1G_03535 | ZP_1938 | RT0436 | [E] |
| ORF0584 | A1C_03415 | RBE_0797 | A1I_05115 | A1E_03015 | RC0629 | RF_0692 | RMA_0639 | RP450 | A1G_03540 | ZP_1937 | RT0437 | [O] |
| ORF0588 | A1C_03445 | RBE_0621 | A1I_04465 | A1E_02995 | RC0634 | RF_0697 | RMA_0643 | RP517 | A1G_03585 | ZP_1933 | RT0503 | [J] |
| ORF0590 | A1C_03455 | RBE_0983 | A1I_03740 | A1E_02990 | RC0636 | RF_0699 | RMA_0646 | RP515 | A1G_03600 | ZP_1930 | RT0501 | [H] |
| ORF0591 | A1C_03465 | RBE_0982 | A1I_03745 | A1E_02985 | RC0637 | RF_0700 | RMA_0647 | RP514 | A1G_03605 | ZP_1929 | RT0500 | [O] |
| ORF0596 | A1C_03480 | RBE_0733 | A1I_04770 | A1E_02970 | RC0647 | RF_0709 | RMA_0653 | RP513 | A1G_03670 | ZP_1923 | RT0498 | [F] |
| ORF0598 | A1C_03485 | RBE_0732 | A1I_04765 | A1E_02965 | RC0651 | RF_0710 | RMA_0657 | RP512 | A1G_03685 | ZP_1920 | RT0497 | [F] |
| ORF0602 | A1C_03530 | RBE_0779 | A1I_05010 | A1E_02955 | RC0656 | RF_0714 | RMA_0663 | RP510 | A1G_03725 | ZP_1917 | RT0495 | [J] |
| ORF0603 | A1C_03535 | RBE_0778 | A1I_05005 | A1E_02950 | RC0657 | RF_0716 | RMA_0664 | RP509 | A1G_03735 | ZP_1916 | RT0494 | [G] |
| ORF0604 | A1C_03540 | RBE_0777 | A1I_05000 | A1E_02945 | RC0658 | RF_0717 | RMA_0665 | RP508 | A1G_03745 | ZP_1915 | RT0493 | [R] |
| ORF0605 | A1C_03545 | RBE_0776 | A1I_04995 | A1E_02940 | RC0659 | RF_0718 | RMA_0666 | RP507 | A1G_03750 | ZP_1914 | RT0492 | [S] |
| ORF0606 | A1C_03550 | RBE_0775 | A1I_04990 | A1E_02935 | RC0660 | RF_0719 | RMA_0667 | RP506 | A1G_03755 | ZP_1913 | RT0491 | [S] |
| ORF0607 | A1C_03555 | RBE_0774 | A1I_04985 | A1E_02930 | RC0661 | RF_0720 | RMA_0668 | RP505 | A1G_03760 | ZP_1912 | RT0490 | [M] |
| ORF0608 | A1C_03560 | RBE_0773 | A1I_04980 | A1E_02925 | RC0663 | RF_0721 | RMA_0669 | RP504 | A1G_03770 | ZP_1911 | RT0489 | [J] |
| ORF0609 | A1C_03565 | RBE_0772 | A1I_04975 | A1E_02920 | RC0664 | RF_0722 | RMA_0670 | RP503 | A1G_03775 | ZP_1910 | RT0488 | [J] |
| ORF0610 | A1C_03570 | RBE_0771 | A1I_04970 | A1E_02915 | RC0665 | RF_0723 | RMA_0671 | RP501 | A1G_03780 | ZP_1909 | RT0487 | [J] |
| ORF0611 | A1C_03575 | RBE_0770 | A1I_04965 | A1E_02910 | RC0666 | RF_0724 | RMA_0672 | RP500 | A1G_03785 | ZP_1908 | RT0486 | [C] |
| ORF0612 | A1C_03580 | RBE_0769 | A1I_04960 | A1E_02905 | RC0667 | RF_0725 | RMA_0673 | RP498 | A1G_03790 | ZP_1907 | RT0485 | [L] |
| ORF0613 | A1C_03595 | RBE_0676 | A1I_04105 | A1E_02900 | RC0668 | RF_0726 | RMA_0674 | RP497 | A1G_03795 | ZP_1906 | RT0484 | [K] |
| ORF0614 | A1C_03600 | RBE_0675 | A1I_04110 | A1E_02895 | RC0670 | RF_0727 | RMA_0675 | RP496 | A1G_03800 | ZP_1905 | RT0483 | [S] |
| ORF0615 | A1C_03605 | RBE_0679 | A1I_04475 | A1E_02890 | RC0671 | RF_0728 | RMA_0676 | RP495 | A1G_03805 | ZP_1904 | RT0482 | [E] |
| ORF0616 | A1C_03610 | RBE_0680 | A1I_04480 | A1E_02885 | RC0672 | RF_0729 | RMA_0677 | RP494 | A1G_03810 | ZP_1903 | RT0481 | [S] |
| ORF0620 | A1C_03625 | RBE_0952 | A1I_03900 | A1E_02870 | RC0678 | RF_0733 | RMA_0681 | RP493 | A1G_03825 | ZP_1899 | RT0479 | [G] |
| ORF0622 | A1C_03640 | RBE_1205 | A1I_01250 | A1E_02845 | RC0783 | RF_0735 | RMA_0683 | RP492 | A1G_03840 | ZP_1897 | RT0478 | [G] |
| ORF0623 | A1C_03645 | RBE_1204 | A1I_01255 | A1E_02840 | RC0782 | RF_0736 | RMA_0684 | RP490 | A1G_03845 | ZP_1896 | RT0476 | [O] |
| ORF0626 | A1C_03665 | RBE_1202 | A1I_01270 | A1E_02820 | RC0779 | RF_0740 | RMA_0687 | RP536 | A1G_03865 | ZP_1893 | RT0524 | [H] |
| ORF0627 | A1C_03670 | RBE_1195 | A1I_01305 | A1E_02815 | RC0778 | RF_0741 | RMA_0688 | RP535 | A1G_03870 | ZP_1892 | RT0523 | [P] |
| ORF0632 | A1C_03700 | RBE_0782 | A1I_05025 | A1E_02785 | RC0773 | RF_0744 | RMA_0690 | RP533 | A1G_03895 | ZP_1889 | RT0521 | [H] |
| ORF0637 | A1C_03745 | RBE_0302 | A1I_06295 | A1E_02765 | RC0766 | RF_0759 | RMA_0700 | RP532 | A1G_03925 | ZP_1884 | RT0519 | [S] |
| ORF0638 | A1C_03750 | RBE_0301 | A1I_06300 | A1E_02760 | RC0765 | RF_0760 | RMA_0701 | RP531 | A1G_03930 | ZP_1883 | RT0518 | [J] |
| ORF0639 | A1C_03755 | RBE_0300 | A1I_06305 | A1E_02755 | RC0764 | RF_0761 | RMA_0702 | RP530 | A1G_03935 | ZP_1882 | RT0517 | [C] |
| ORF0640 | A1C_03760 | RBE_0298 | A1I_06315 | A1E_02750 | RC0762 | RF_0762 | RMA_0703 | RP529 | A1G_03940 | ZP_1881 | RT0516 | [J] |
| ORF0643 | A1C_03765 | RBE_0295 | A1I_06330 | A1E_02745 | RC0761 | RF_0763 | RMA_0704 | RP528 | A1G_03945 | ZP_3113 | RT0515 | [L] |
| ORF0644 | A1C_03770 | RBE_0294 | A1I_06335 | A1E_02740 | RC0760 | RF_0764 | RMA_0705 | RP527 | A1G_03950 | ZP_3112 | RT0514 | [QR] |
| ORF0645 | A1C_03775 | RBE_0737 | A1I_04790 | A1E_02735 | RC0759 | RF_0765 | RMA_0706 | RP526 | A1G_03955 | ZP_3111 | RT0513 | [K] |
| ORF0647 | A1C_03790 | RBE_0735 | A1I_04780 | A1E_02725 | RC0757 | RF_0767 | RMA_0708 | RP524 | A1G_03965 | ZP_3109 | RT0511 | [L] |
| ORF0662 | A1C_04245 | RBE_0413 | A1I_05675 | A1E_02580 | RC0748 | RF_0770 | RMA_0758 | RP522 | A1G_04215 | ZP_3102 | RT0509 | [F] |
| ORF0664 | A1C_04235 | RBE_0411 | A1I_05685 | A1E_02570 | RC0746 | RF_0772 | RMA_0760 | RP520 | A1G_04200 | ZP_3100 | RT0507 | [OU] |
| ORF0665 | A1C_04195 | RBE_0731 | A1I_04760 | A1E_02555 | RC0744 | RF_0785 | RMA_0766 | RP452 | A1G_04195 | ZP_3099 | RT0439 | [C] |
| ORF0671 | A1C_03975 | RBE_0780 | A1I_05015 | A1E_02320 | RC0736 | RF_0849 | RMA_0829 | RP489 | A1G_04155 | ZP_3092 | RT0475 | No hit |
| ORF0675 | A1C_03965 | RBE_0948 | A1I_03925 | A1E_02330 | RC0731 | RF_0846 | RMA_0824 | RP487 | A1G_04130 | ZP_3088 | RT0474 | [E] |
| ORF0676 | A1C_03960 | RBE_0947 | A1I_03930 | A1E_02335 | RC0730 | RF_0845 | RMA_0823 | RP486 | A1G_04125 | ZP_3087 | RT0473 | [E] |
| ORF0677 | A1C_03955 | RBE_0946 | A1I_03935 | A1E_02340 | RC0729 | RF_0844 | RMA_0822 | RP485 | A1G_04120 | ZP_3086 | RT0472 | [C] |
| ORF0678 | A1C_03950 | RBE_0945 | A1I_03940 | A1E_02345 | RC0728 | RF_0843 | RMA_0821 | RP484 | A1G_04115 | ZP_3085 | RT0471 | [S] |
| ORF0681 | A1C_03915 | RBE_0916 | A1I_04095 | A1E_02355 | RC0725 | RF_0840 | RMA_0819 | RP482 | A1G_04105 | ZP_3082 | RT0469 | [E] |
| ORF0682 | A1C_03830 | RBE_0717 | A1I_04680 | A1E_02360 | RC0724 | RF_0839 | RMA_0818 | RP479 | A1G_04100 | ZP_3081 | RT0466 | [H] |
| ORF0683 | A1C_03835 | RBE_0718 | A1I_04685 | A1E_02365 | RC0723 | RF_0838 | RMA_0817 | RP478 | A1G_04095 | ZP_3080 | RT0465 | No hit |
| ORF0684 | A1C_03840 | RBE_0721 | A1I_04700 | A1E_02370 | RC0722 | RF_0837 | RMA_0816 | RP477 | A1G_04090 | ZP_3079 | RT0464 | [C] |
| ORF0688 | A1C_03850 | RBE_0723 | A1I_04710 | A1E_02380 | RC0718 | RF_0834 | RMA_0812 | RP475 | A1G_04070 | ZP_3077 | RT0462 | [GEPR] |
| ORF0690 | A1C_03865 | RBE_0726 | A1I_04725 | A1E_02390 | RC0716 | RF_0831 | RMA_0810 | RP474 | A1G_04055 | ZP_3075 | RT0461 | No hit |
| ORF0693 | A1C_03880 | RBE_0787 | A1I_05050 | A1E_02405 | RC0713 | RF_0828 | RMA_0806 | RP471 | A1G_04040 | ZP_3072 | RT0458 | [R] |
| ORF0695 | A1C_03895 | RBE_0788 | A1I_05055 | A1E_02415 | RC0712 | RF_0826 | RMA_0805 | RP470 | A1G_04030 | ZP_3071 | RT0457 | [N] |
| ORF0696 | A1C_03900 | RBE_0789 | A1I_05065 | A1E_02420 | RC0711 | RF_0825 | RMA_0804 | RP469 | A1G_04025 | ZP_3070 | RT0456 | [I] |
| ORF0697 | A1C_03905 | RBE_0790 | A1I_05070 | A1E_02425 | RC0710 | RF_0824 | RMA_0803 | RP468 | A1G_04020 | ZP_3069 | RT0455 | [J] |
| ORF0699 | A1C_03825 | RBE_0346 | A1I_06040 | A1E_02435 | RC0706 | RF_0817 | RMA_0799 | RP466 | A1G_04275 | ZP_3066 | RT0453 | [H] |
| ORF0704 | A1C_04115 | RBE_0688 | A1I_04520 | A1E_02470 | RC0698 | RF_0811 | RMA_0790 | RP464 | A1G_04315 | ZP_3058 | RT0451 | [R] |
| ORF0705 | A1C_04120 | RBE_0689 | A1I_04525 | A1E_02475 | RC0697 | RF_0810 | RMA_0789 | RP463 | A1G_04320 | ZP_3057 | RT0450 | [S] |
| ORF0706 | A1C_04125 | RBE_0690 | A1I_04530 | A1E_02480 | RC0696 | RF_0809 | RMA_0788 | RP462 | A1G_04325 | ZP_3056 | RT0449 | [J] |
| ORF0707 | A1C_04130 | RBE_0893 | A1I_02425 | A1E_02485 | RC0694 | RF_0808 | RMA_0787 | RP461 | A1G_04335 | ZP_3054 | RT0448 | [H] |
| ORF0708 | A1C_04135 | RBE_0578 | A1I_03305 | A1E_02490 | RC0693 | RF_0807 | RMA_0786 | RP460 | A1G_04340 | ZP_3053 | RT0447 | [C] |
| ORF0709 | A1C_04140 | RBE_0577 | A1I_03300 | A1E_02495 | RC0692 | RF_0806 | RMA_0785 | RP459 | A1G_04345 | ZP_3052 | RT0446 | [QR] |
| ORF0713 | A1C_04150 | RBE_0574 | A1I_03280 | A1E_02500 | RC0687 | RF_0804 | RMA_0780 | RP458 | A1G_04370 | ZP_3047 | RT0445 | No hit |
| ORF0714 | A1C_04155 | RBE_0573 | A1I_03275 | A1E_02505 | RC0686 | RF_0803 | RMA_0779 | RP457 | A1G_04375 | ZP_3046 | RT0444 | [M] |
| ORF0716 | A1C_04170 | RBE_0569 | A1I_03245 | A1E_02515 | RC0682 | RF_0800 | RMA_0776 | RP456 | A1G_04395 | ZP_3043 | RT0443 | [J] |
| ORF0717 | A1C_04175 | RBE_0568 | A1I_03240 | A1E_02520 | RC0681 | RF_0799 | RMA_0775 | RP455 | A1G_04400 | ZP_3042 | RT0442 | [S] |
| ORF0718 | A1C_04180 | RBE_0567 | A1I_03235 | A1E_02525 | RC0680 | RF_0798 | RMA_0774 | RP454 | A1G_04405 | ZP_3041 | RT0441 | [M] |
| ORF0726 | A1C_03985 | RBE_0781 | A1I_05020 | A1E_02295 | RC0796 | RF_0854 | RMA_0834 | RP537 | A1G_04480 | ZP_3037 | RT0526 | [C] |
| ORF0727 | A1C_03990 | RBE_0919 | A1I_04070 | A1E_02290 | RC0797 | RF_0855 | RMA_0835 | RP538 | A1G_04485 | ZP_3036 | RT0527 | No hit |
| ORF0728 | A1C_03995 | RBE_0920 | A1I_04065 | A1E_02285 | RC0798 | RF_0856 | RMA_0836 | RP539 | A1G_04490 | ZP_3035 | RT0528 | [H] |
| ORF0732 | A1C_04000 | RBE_0925 | A1I_04040 | A1E_02275 | RC0800 | RF_0858 | RMA_0838 | RP540 | A1G_04510 | ZP_3032 | RT0529 | [L] |
| ORF0733 | A1C_04005 | RBE_0926 | A1I_04035 | A1E_02270 | RC0801 | RF_0859 | RMA_0839 | RP541 | A1G_04515 | ZP_3031 | RT0530 | [H] |
| ORF0734 | A1C_04010 | RBE_0929 | A1I_04020 | A1E_02265 | RC0803 | RF_0860 | RMA_0840 | RP542 | A1G_04525 | ZP_3030 | RT0531 | [L] |
| ORF0736 | A1C_04025 | RBE_0321 | A1I_06190 | A1E_02250 | RC0807 | RF_0863 | RMA_0845 | RP544 | A1G_04550 | ZP_3027 | RT0532 | [J] |
| ORF0738 | A1C_04035 | RBE_0320 | A1I_06195 | A1E_02240 | RC0809 | RF_0865 | RMA_0847 | RP545 | A1G_04560 | ZP_3025 | RT0533 | [L] |
| ORF0739 | A1C_04040 | RBE_0316 | A1I_06215 | A1E_02235 | RC0810 | RF_0866 | RMA_0848 | RP546 | A1G_04565 | ZP_3024 | RT0534 | [O] |
| ORF0740 | A1C_04045 | RBE_0291 | A1I_06350 | A1E_02230 | RC0811 | RF_0867 | RMA_0849 | RP547 | A1G_04570 | ZP_3023 | RT0535 | No hit |
| ORF0741 | A1C_04050 | RBE_0292 | A1I_06345 | A1E_02225 | RC0812 | RF_0868 | RMA_0850 | RP548m | A1G_04575 | ZP_3022 | RT0536 | [L] |
| ORF0742 | A1C_04055 | RBE_0333 | A1I_06120 | A1E_02220 | RC0814 | RF_0869 | RMA_0851 | RP551 | A1G_04585 | ZP_3020 | RT0537 | [O] |
| ORF0744 | A1C_04065 | RBE_0613 | A1I_03495 | A1E_02210 | RC0816 | RF_0871 | RMA_0853 | RP552 | A1G_04595 | ZP_3018 | RT0539 | [J] |
| ORF0745 | A1C_04070 | RBE_0612 | A1I_03490 | A1E_02205 | RC0817 | RF_0872 | RMA_0854 | RP553 | A1G_04600 | ZP_3017 | RT0540 | [K] |
| ORF0746 | A1C_04075 | RBE_0611 | A1I_03485 | A1E_02200 | RC0818 | RF_0873 | RMA_0855 | RP554 | A1G_04605 | ZP_3016 | RT0541 | [S] |
| ORF0749 | A1C_04090 | RBE_0608 | A1I_03470 | A1E_02195 | RC0822 | RF_0876 | RMA_0859 | RP555 | A1G_04625 | ZP_3013 | RT0543 | [J] |
| ORF0771 | A1C_04350 | RBE_0625 | A1I_04440 | A1E_02120 | RC0848 | RF_0893 | RMA_0886 | RP561 | A1G_04705 | ZP_2992 | RT0548 | [HC] |
| ORF0773 | A1C_04360 | RBE_0312 | A1I_06235 | A1E_02110 | RC0849 | RF_0895 | RMA_0888 | RP562 | A1G_04715 | ZP_2991 | RT0550 | [T] |
| ORF0774 | A1C_04365 | RBE_0313 | A1I_06230 | A1E_02105 | RC0850 | RF_0896 | RMA_0889 | RP563 | A1G_04720 | ZP_2990 | RT0551 | [S] |
| ORF0775 | A1C_04370 | RBE_1221 | A1I_01170 | A1E_02100 | RC0851 | RF_0897 | RMA_0890 | RP564 | A1G_04725 | ZP_2989 | RT0552 | No hit |
| ORF0776 | A1C_04385 | RBE_1223 | A1I_01180 | A1E_02085 | RC0852 | RF_0900 | RMA_0891 | RP565 | A1G_04730 | ZP_2988 | RT0553 | [M] |
| ORF0778 | A1C_04395 | RBE_0542 | A1I_03100 | A1E_02075 | RC0855 | RF_0902 | RMA_0893 | RP567 | A1G_04745 | ZP_2986 | RT0555 | [M] |
| ORF0779 | A1C_04400 | RBE_0541 | A1I_03095 | A1E_02070 | RC0856 | RF_0903 | RMA_0894 | RP568 | A1G_04750 | ZP_2985 | RT0556 | [S] |
| ORF0780 | A1C_04405 | RBE_0540 | A1I_03090 | A1E_02065 | RC0857 | RF_0904 | RMA_0895 | RP569 | A1G_04755 | ZP_2984 | RT0557 | [M] |
| ORF0781 | A1C_04410 | RBE_0539 | A1I_03085 | A1E_02060 | RC0858 | RF_0905 | RMA_0896 | RP570 | A1G_04760 | ZP_2983 | RT0558 | [S] |
| ORF0783 | A1C_04415 | RBE_0751 | A1I_04865 | A1E_02030 | RC0862 | RF_0906 | RMA_0899 | RP571 | A1G_04770 | ZP_2982 | RT0559 | [ER] |
| ORF0784 | A1C_04430 | RBE_0614 | A1I_03500 | A1E_02035 | RC0864 | RF_0912 | RMA_0901 | RP572 | A1G_04780 | ZP_2980 | RT0560 | [L] |
| ORF0785 | A1C_04435 | RBE_0691 | A1I_04535 | A1E_02040 | RC0865 | RF_0913 | RMA_0902 | RP573 | A1G_04785 | ZP_2979 | RT0561 | [S] |
| ORF0796 | A1C_04485 | RBE_0897 | A1I_02405 | A1E_01990 | RC0879 | RF_0941 | RMA_0912 | RP575 | A1G_04855 | ZP_2968 | RT0564 | [U] |
| ORF0797 | A1C_04490 | RBE_0898 | A1I_02400 | A1E_01985 | RC0880 | RF_0942 | RMA_0913 | RP576 | A1G_04860 | ZP_2967 | RT0565 | [O] |
| ORF0798 | A1C_04495 | RBE_0992 | A1I_03695 | A1E_01980 | RC0881 | RF_0943 | RMA_0914 | RP577 | A1G_04865 | ZP_2966 | RT0566 | [I] |
| ORF0799 | A1C_04500 | RBE_0993 | A1I_03690 | A1E_01975 | RC0882 | RF_0944 | RMA_0915 | RP578 | A1G_04870 | ZP_2965 | RT0567 | [K] |
| ORF0800 | A1C_04505 | RBE_0994 | A1I_03685 | A1E_01970 | RC0883 | RF_0945 | RMA_0916 | RP579 | A1G_04875 | ZP_2964 | RT0568 | [M] |
| ORF0801 | A1C_04510 | RBE_0995 | A1I_03680 | A1E_01965 | RC0884 | RF_0947 | RMA_0917 | RP580 | A1G_04880 | ZP_2963 | RT0569 | [L] |
| ORF0802 | A1C_04515 | RBE_0996 | A1I_03675 | A1E_01960 | RC0885 | RF_0948 | RMA_0918 | RP581 | A1G_04885 | ZP_2962 | RT0570 | [G] |
| ORF0803 | A1C_04520 | RBE_0999 | A1I_03650 | A1E_01940 | RC0886 | RF_0951 | RMA_0919 | RP583 | A1G_04890 | ZP_2961 | RT0572 | [P] |
| ORF0809 | A1C_04560 | RBE_0701 | A1I_04600 | A1E_01935 | RC0893 | RF_0958 | RMA_0925 | RP585 | A1G_04935 | ZP_2956 | RT0574 | [U] |
| ORF0810 | A1C_04565 | RBE_0700 | A1I_04595 | A1E_01930 | RC0894 | RF_0959 | RMA_0926 | RP586 | A1G_04940 | ZP_2955 | RT0575 | [U] |
| ORF0811 | A1C_04570 | RBE_0699 | A1I_04590 | A1E_01925 | RC0895 | RF_0960 | RMA_0927 | RP587 | A1G_04945 | ZP_2954 | RT0576 | [R] |
| ORF0813 | A1C_04575 | RBE_0698 | A1I_04580 | A1E_01920 | RC0896 | RF_0961 | RMA_0929 | RP588 | A1G_04950 | ZP_2953 | RT0577 | [O] |
| ORF0814 | A1C_04580 | RBE_0697 | A1I_04575 | A1E_01915 | RC0897 | RF_0962 | RMA_0930 | RP589 | A1G_04955 | ZP_2952 | RT0578 | [C] |
| ORF0815 | A1C_04585 | RBE_0696 | A1I_04570 | A1E_01905 | RC0898 | RF_0963 | RMA_0931 | RP590 | A1G_04960 | ZP_2951 | RT0579 | [R] |
| ORF0816 | A1C_04590 | RBE_0694 | A1I_04560 | A1E_01900 | RC0899 | RF_0964 | RMA_0932 | RP591 | A1G_04965 | ZP_2950 | RT0580 | No hit |
| ORF0825 | A1C_04660 | RBE_0854 | A1I_02650 | A1E_01855 | RC0910 | RF_0370 | RMA_0942 | RP595 | A1G_05025 | ZP_2938 | RT0583 | [M] |
| ORF0826 | A1C_04665 | RBE_0853 | A1I_02655 | A1E_01850 | RC0911 | RF_0369 | RMA_0943 | RP596 | A1G_05030 | ZP_2937 | RT0584 | [M] |
| ORF0827 | A1C_04670 | RBE_0852 | A1I_02660 | A1E_01845 | RC0912 | RF_0368 | RMA_0944 | RP597 | A1G_05035 | ZP_2936 | RT0585 | [M] |
| ORF0828 | A1C_04675 | RBE_0851 | A1I_02665 | A1E_01840 | RC0913 | RF_0367 | RMA_0945 | RP598 | A1G_05040 | ZP_2935 | RT0586 | [LK] |
| ORF0829 | A1C_04680 | RBE_0850 | A1I_02670 | A1E_01835 | RC0914 | RF_0366 | RMA_0946 | RP599 | A1G_05045 | ZP_2934 | RT0587 | [S] |
| ORF0830 | A1C_04685 | RBE_0849 | A1I_02675 | A1E_01830 | RC0915 | RF_0365 | RMA_0947 | RP600 | A1G_05050 | ZP_2933 | RT0588 | [P] |
| ORF0831 | A1C_04690 | RBE_0848 | A1I_02685 | A1E_01825 | RC0916 | RF_0364 | RMA_0948 | RP601 | A1G_05055 | ZP_2932 | RT0589 | [L] |
| ORF0835 | A1C_04710 | RBE_0843 | A1I_02710 | A1E_01815 | RC0922 | RF_0360 | RMA_0952 | RP602 | A1G_05085 | ZP_2926 | RT0590 | [R] |
| ORF0841 | A1C_04730 | RBE_0857 | A1I_02620 | A1E_01775 | RC0928 | RF_0355 | RMA_0958 | RP604 | A1G_05115 | ZP_2919 | RT0592 | [J] |
| ORF0843 | A1C_04745 | RBE_0861 | A1I_02595 | A1E_01765 | RC0931 | RF_0353 | RMA_0960 | RP605 | A1G_05130 | ZP_2916 | RT0593 | [J] |
| ORF0844 | A1C_04750 | RBE_0862 | A1I_02590 | A1E_01760 | RC0932 | RF_0352 | RMA_0961 | RP606 | A1G_05135 | ZP_2915 | RT0594 | [J] |
| ORF0845 | A1C_04755 | RBE_0863 | A1I_02585 | A1E_01755 | RC0933 | RF_0351 | RMA_0962 | RP607 | A1G_05140 | ZP_2914 | RT0595 | [O] |
| ORF0846 | A1C_04765 | RBE_0864 | A1I_02580 | A1E_01750 | RC0934 | RF_0350 | RMA_0963 | RP608 | A1G_05145 | ZP_2913 | RT0596 | [J] |
| ORF0847 | A1C_04770 | RBE_0865 | A1I_02575 | A1E_01745 | RC0935 | RF_0349 | RMA_0964 | RP609 | A1G_05150 | ZP_2912 | RT0597 | [J] |
| ORF0848 | A1C_04775 | RBE_0866 | A1I_02570 | A1E_01740 | RC0936 | RF_0348 | RMA_0965 | RP610 | A1G_05155 | ZP_2911 | RT0598 | [J] |
| ORF0856 | A1C_04820 | RBE_0470 | A1I_05325 | A1E_01705 | RC0948 | RF_0427 | RMA_0977 | RP614 | A1G_05210 | ZP_2904 | RT0603 | [T] |
| ORF0857 | A1C_04825 | RBE_0454 | A1I_05430 | A1E_01695 | RC0949 | RF_0426 | RMA_0978 | RP615 | A1G_05220 | ZP_2903 | RT0604 | [J] |
| ORF0858 | A1C_04830 | RBE_0453 | A1I_05435 | A1E_01690 | RC0950 | RF_0425 | RMA_0979 | RP616 | A1G_05225 | ZP_2902 | RT0605 | [S] |
| ORF0859 | A1C_04840 | RBE_0602 | A1I_03430 | A1E_01685 | RC0953 | RF_0423 | RMA_0982 | RP617 | A1G_05240 | ZP_2900 | RT0606 | [J] |
| ORF0862 | A1C_04855 | RBE_0469 | A1I_05330 | A1E_01675 | RC0959 | RF_0419 | RMA_0985 | RP618 | A1G_05265 | ZP_2896 | RT0608 | [I] |
| ORF0863 | A1C_04860 | RBE_0467 | A1I_05340 | A1E_01660 | RC0960 | RF_0418 | RMA_0986 | RP619 | A1G_05270 | ZP_2895 | RT0609 | [I] |
| ORF0865 | A1C_04875 | RBE_0465 | A1I_05365 | A1E_01650 | RC0962 | RF_0417 | RMA_0988 | RP620 | A1G_05280 | ZP_2893 | RT0611 | [IQ] |
| ORF0868 | A1C_04890 | RBE_0456 | A1I_05420 | A1E_01640 | RC0965 | RF_0414 | RMA_0991 | RP622 | A1G_05300 | ZP_2890 | RT0613 | [H] |
| ORF0869 | A1C_04895 | RBE_0457 | A1I_05405 | A1E_01635 | RC0966 | RF_0413 | RMA_0992 | RP623 | A1G_05305 | ZP_2889 | RT0614 | [J] |
| ORF0871 | A1C_04925 | RBE_0590 | A1I_03365 | A1E_01620 | RC0968 | RF_0409 | RMA_0997 | RP626 | A1G_05320 | ZP_2887 | RT0617 | [O] |
| ORF0872 | A1C_04930 | RBE_0591 | A1I_03370 | A1E_01615 | RC0969 | RF_0408 | RMA_0998 | RP627 | A1G_05325 | ZP_2886 | RT0618 | [O] |
| ORF0877 | A1C_04950 | RBE_0607 | A1I_03465 | A1E_01605 | RC0975 | RF_0403 | RMA_1006 | RP628 | A1G_05365 | ZP_2881 | RT0619 | [J] |
| ORF0878 | A1C_04955 | RBE_0606 | A1I_03460 | A1E_01600 | RC0977 | RF_0402 | RMA_1007 | RP629 | A1G_05370 | ZP_2880 | RT0620 | [O] |
| ORF0879 | A1C_04960 | RBE_0605 | A1I_03450 | A1E_01595 | RC0978 | RF_0401 | RMA_1008 | RP630 | A1G_05375 | ZP_2879 | RT0621 | [R] |
| ORF0880 | A1C_04965 | RBE_0536 | A1I_03070 | A1E_01590 | RC0979 | RF_0400 | RMA_1009 | RP631 | A1G_05380 | ZP_2878 | RT0622 | [L] |
| ORF0886 | A1C_04975 | RBE_1036 | A1I_02170 | A1E_04240 | RC0980 | RF_0304 | RMA_1014 | RP633 | A1G_05425 | ZP_2875 | RT0625 | [J] |
| ORF0887 | A1C_04980 | RBE_1037 | A1I_02165 | A1E_04245 | RC0981 | RF_0303 | RMA_1015 | RP634 | A1G_05430 | ZP_2874 | RT0626 | [J] |
| ORF0888 | A1C_04985 | RBE_1038 | A1I_02160 | A1E_04250 | RC0982 | RF_0302 | RMA_1016 | RP635 | A1G_05435 | ZP_2873 | RT0627 | [K] |
| ORF0889 | A1C_04990 | RBE_1039 | A1I_02155 | A1E_04255 | RC0983 | RF_0301 | RMA_1017 | RP636 | A1G_05440 | ZP_2872 | RT0628 | [J] |
| ORF0890 | A1C_04995 | RBE_1040 | A1I_02150 | A1E_04260 | RC0984 | RF_0300 | RMA_1018 | RP637 | A1G_05445 | ZP_2871 | RT0629 | [J] |
| ORF0892 | A1C_05005 | RBE_1042 | A1I_02135 | A1E_04270 | RC0986 | RF_0298 | RMA_1020 | RP639 | A1G_05455 | ZP_2869 | RT0631 | [U] |
| ORF0893 | A1C_05010 | RBE_1043 | A1I_02130 | A1E_04275 | RC0987 | RF_0297 | RMA_1021 | RP640 | A1G_05460 | ZP_2868 | RT0632 | [J] |
| ORF0894 | A1C_05015 | RBE_1044 | A1I_02125 | A1E_04280 | RC0988 | RF_0296 | RMA_1022 | RP641 | A1G_05465 | ZP_2867 | RT0633 | [J] |
| ORF0895 | A1C_05020 | RBE_1045 | A1I_02120 | A1E_04285 | RC0989 | RF_0295 | RMA_1023 | RP642 | A1G_05470 | ZP_2866 | RT0634 | [J] |
| ORF0896 | A1C_05025 | RBE_1046 | A1I_02115 | A1E_04290 | RC0990 | RF_0294 | RMA_1024 | RP643 | A1G_05475 | ZP_2865 | RT0635 | [J] |
| ORF0897 | A1C_05030 | RBE_1047 | A1I_02110 | A1E_04295 | RC0991 | RF_0293 | RMA_1025 | RP644 | A1G_05480 | ZP_2864 | RT0636 | [J] |
| ORF0898 | A1C_05035 | RBE_1048 | A1I_02105 | A1E_04300 | RC0992 | RF_0292 | RMA_1026 | RP645 | A1G_05485 | ZP_2863 | RT0637 | [J] |
| ORF0899 | A1C_05040 | RBE_1049 | A1I_02100 | A1E_04305 | RC0993 | RF_0291 | RMA_1027 | RP646 | A1G_05490 | ZP_2862 | RT0638 | [J] |
| ORF0900 | A1C_05045 | RBE_1050 | A1I_02095 | A1E_04310 | RC0994 | RF_0290 | RMA_1028 | RP647 | A1G_05495 | ZP_2861 | RT0639 | [J] |
| ORF0902 | A1C_05055 | RBE_1052 | A1I_02085 | A1E_04320 | RC0996 | RF_0288 | RMA_1030 | RP649 | A1G_05505 | ZP_2859 | RT0641 | [J] |
| ORF0903 | A1C_05060 | RBE_1053 | A1I_02080 | A1E_04325 | RC0997 | RF_0287 | RMA_1031 | RP650 | A1G_05510 | ZP_2858 | RT0642 | [J] |
| ORF0904 | A1C_05065 | RBE_1054 | A1I_02075 | A1E_04330 | RC0998 | RF_0286 | RMA_1032 | RP651 | A1G_05515 | ZP_2857 | RT0643 | [J] |
| ORF0905 | A1C_05070 | RBE_1055 | A1I_02070 | A1E_04335 | RC0999 | RF_0285 | RMA_1033 | RP652 | A1G_05520 | ZP_2856 | RT0644 | [J] |
| ORF0906 | A1C_05075 | RBE_1056 | A1I_02065 | A1E_04340 | RC1000 | RF_0284 | RMA_1034 | RP653 | A1G_05525 | ZP_2855 | RT0645 | [J] |
| ORF0907 | A1C_05080 | RBE_1057 | A1I_02060 | A1E_04345 | RC1001 | RF_0283 | RMA_1035 | RP654 | A1G_05530 | ZP_2854 | RT0646 | [J] |
| ORF0908 | A1C_05085 | RBE_1058 | A1I_02055 | A1E_04350 | RC1002 | RF_0282 | RMA_1036 | RP655 | A1G_05535 | ZP_2853 | RT0647 | [J] |
| ORF0909 | A1C_05090 | RBE_1059 | A1I_02050 | A1E_04355 | RC1003 | RF_0281 | RMA_1037 | RP656 | A1G_05540 | ZP_2852 | RT0648 | [J] |
| ORF0910 | A1C_05095 | RBE_1060 | A1I_02045 | A1E_04360 | RC1004 | RF_0280 | RMA_1038 | RP657 | A1G_05545 | ZP_2851 | RT0649 | [J] |
| ORF0911 | A1C_05100 | RBE_1061 | A1I_02040 | A1E_04365 | RC1005 | RF_0279 | RMA_1039 | RP658 | A1G_05550 | ZP_2850 | RT0650 | [J] |
| ORF0912 | A1C_05105 | RBE_1062 | A1I_02035 | A1E_04370 | RC1006 | RF_0278 | RMA_1040 | RP659 | A1G_05555 | ZP_2849 | RT0651 | [J] |
| ORF0913 | A1C_05110 | RBE_1063 | A1I_02030 | A1E_04375 | RC1007 | RF_0277 | RMA_1041 | RP660 | A1G_05560 | ZP_2848 | RT0652 | [J] |
| ORF0914 | A1C_05115 | RBE_1064 | A1I_02025 | A1E_04380 | RC1008 | RF_0276 | RMA_1042 | RP661 | A1G_05565 | ZP_2847 | RT0653 | [J] |
| ORF0915 | A1C_05120 | RBE_1065 | A1I_02020 | A1E_04385 | RC1009 | RF_0275 | RMA_1043 | RP664 | A1G_05570 | ZP_2846 | RT0656 | [J] |
| ORF0917 | A1C_05130 | RBE_1067 | A1I_02010 | A1E_04395 | RC1012 | RF_0273 | RMA_1045 | RP665 | A1G_05585 | ZP_2844 | RT0657 | [C] |
| ORF0920 | A1C_05145 | RBE_1071 | A1I_01985 | A1E_04405 | RC1015 | RF_0270 | RMA_1048 | RP666 | A1G_05600 | ZP_2842 | RT0658 | [D] |
| ORF0923 | A1C_05170 | RBE_1073 | A1I_01975 | A1E_04420 | RC1018 | RF_0267 | RMA_1051 | RP667 | A1G_05615 | ZP_2839 | RT0659 | [O] |
| ORF0925 | A1C_05185 | RBE_1076 | A1I_01955 | A1E_04425 | RC1020 | RF_0264 | RMA_1053 | RP669 | A1G_05625 | ZP_2837 | RT0661 | [LKJ] |
| ORF0926 | A1C_05190 | RBE_1077 | A1I_01950 | A1E_04430 | RC1021 | RF_0262 | RMA_1054 | RP670 | A1G_05630 | ZP_2836 | RT0662 | [K] |
| ORF0928 | A1C_05200 | RBE_0204 | A1I_06805 | A1E_04455 | RC1022 | RF_0256 | RMA_1056 | RP672 | A1G_05635 | ZP_2835 | RT0666 | [J] |
| ORF0929 | A1C_05205 | RBE_0210 | A1I_06775 | A1E_04460 | RC1023 | RF_0255 | RMA_1057 | RP673 | A1G_05640 | ZP_2834 | RT0667 | [O] |
| ORF0930 | A1C_05210 | RBE_0209 | A1I_06780 | A1E_04465 | RC1024 | RF_0254 | RMA_1058 | RP674 | A1G_05645 | ZP_2833 | RT0668 | [M] |
| ORF0932 | A1C_05220 | RBE_0208 | A1I_06785 | A1E_04470 | RC1026 | RF_0253 | RMA_1059 | RP675 | A1G_05650 | ZP_2832 | RT0669 | [L] |
| ORF0934 | A1C_05230 | RBE_0205 | A1I_06800 | A1E_04480 | RC1028 | RF_0250 | RMA_1063 | RP676 | A1G_05670 | ZP_2831 | RT0671 | [L] |
| ORF0936 | A1C_05240 | RBE_0212 | A1I_06755 | A1E_04485 | RC1030 | RF_0248 | RMA_1064 | RP677 | A1G_05685 | ZP_2830 | RT0673 | [R] |
| ORF0940 | A1C_05260 | RBE_0249 | A1I_06735 | A1E_04505 | RC1036 | RF_0244 | RMA_1068 | RP678 | A1G_05725 | ZP_2826 | RT0674 | [R] |
| ORF0941 | A1C_05265 | RBE_0248 | A1I_06730 | A1E_04510 | RC1037 | RF_0243 | RMA_1069 | RP680 | A1G_05735 | ZP_2825 | RT0675 | [H] |
| ORF0950 | A1C_05325 | RBE_0239 | A1I_06685 | A1E_04535 | RC1045 | RF_0236 | RMA_1080 | RP682 | A1G_05805 | ZP_2817 | RT0677 | [L] |
| ORF0951 | A1C_05330 | RBE_0238 | A1I_06680 | A1E_04540 | RC1046 | RF_0235 | RMA_1081 | RP683 | A1G_05810 | ZP_2816 | RT0678 | [J] |
| ORF0952 | A1C_05335 | RBE_0237 | A1I_06675 | A1E_04545 | RC1047 | RF_0234 | RMA_1082 | RP684 | A1G_05815 | ZP_2815 | RT0679 | [F] |
| ORF0953 | A1C_05340 | RBE_0218 | A1I_06565 | A1E_04550 | RC1048 | RF_0233 | RMA_1083 | RP685 | A1G_05820 | ZP_2814 | RT0680 | [GEPR] |
| ORF0954 | A1C_05345 | RBE_0407 | A1I_05710 | A1E_04555 | RC1049 | RF_0231 | RMA_1084 | RP686 | A1G_05825 | ZP_2813 | RT0681 | [H] |
| ORF0957 | A1C_05365 | RBE_0509 | A1I_02925 | A1E_04565 | RC1053 | RF_0229 | RMA_1087 | RP687 | A1G_05850 | ZP_2810 | RT0682 | [J] |
| ORF0962 | A1C_05400 | RBE_0502 | A1I_02890 | A1E_04580 | RC1057 | RF_0225 | RMA_1093 | RP690 | A1G_05870 | ZP_2805 | RT0684 | [S] |
| ORF0974 | A1C_05445 | RBE_0499 | A1I_02875 | A1E_04600 | RC1068 | RF_0219 | RMA_1104 | RP692 | A1G_05925 | ZP_2795 | RT0687 | [O] |
| ORF0975 | A1C_05450 | RBE_0498 | A1I_02870 | A1E_04605 | RC1069 | RF_0218 | RMA_1105 | RP693 | A1G_05930 | ZP_2794 | RT0688 | [J] |
| ORF0976 | A1C_05455 | RBE_0496 | A1I_02865 | A1E_04610 | RC1070 | RF_0217 | RMA_1106 | RP694 | A1G_05935 | ZP_2793 | RT0689 | [R] |
| ORF0978 | A1C_05475 | RBE_0492 | A1I_02845 | A1E_04615 | RC1073 | RF_0214 | RMA_1108 | RP696 | A1G_05950 | ZP_2790 | RT0691 | [V] |
| ORF0979 | A1C_05480 | RBE_0202 | A1I_06820 | A1E_04620 | RC1074 | RF_0213 | RMA_1109 | RP697 | A1G_05955 | ZP_2789 | RT0692 | No hit |
| ORF0980 | A1C_05485 | RBE_0201 | A1I_06830 | A1E_04625 | RC1075 | RF_0212 | RMA_1110 | RP698 | A1G_05960 | ZP_2788 | RT0693 | [GEPR] |
| ORF0982 | A1C_05495 | RBE_0199 | A1I_06845 | A1E_04645 | RC1077 | RF_0210 | RMA_1112 | RP700 | A1G_05970 | ZP_2786 | RT0695 | [V] |
| ORF0983 | A1C_05500 | RBE_0198 | A1I_06850 | A1E_04650 | RC1078 | RF_0209 | RMA_1113 | RP701 | A1G_05975 | ZP_2785 | RT0696 | No hit |
| ORF0990 | A1C_05535 | RBE_0184 | A1I_06950 | A1E_04675 | RC1085 | RF_0205 | RMA_1118 | RP704 | A1G_06030 | ZP_2779 | RT0699 | [U] |
| ORF0992 | A1C_05545 | RBE_0182 | A1I_06960 | A1E_04685 | RC1086 | RF_0203 | RMA_1121 | RP706 | A1G_06040 | ZP_2778 | RT0701 | [G] |
| ORF0997 | A1C_05570 | RBE_0186 | A1I_06940 | A1E_04690 | RC1091 | RF_0197 | RMA_1125 | RP718 | A1G_06065 | ZP_2773 | RT0704 | [M] |
| ORF0998 | A1C_05575 | RBE_0185 | A1I_06945 | A1E_04695 | RC1092 | RF_0196 | RMA_1126 | RP719 | A1G_06070 | ZP_2772 | RT0705 | [M] |
| ORF1003 | A1C_05595 | RBE_0193 | A1I_06890 | A1E_04715 | RC1096 | RF_0192 | RMA_1131 | RP720 | A1G_06085 | ZP_2769 | RT0706 | [L] |
| ORF1005 | A1C_05610 | RBE_1258 | A1I_00995 | A1E_04725 | RC1097 | RF_0191 | RMA_1133 | RP721 | A1G_06095 | ZP_2768 | RT0707 | [J] |
| ORF1014 | A1C_05890 | RBE_0368 | A1I_05930 | A1E_01135 | RC1106 | RF_0182 | RMA_1143 | RP724 | A1G_06135 | ZP_2763 | RT0711 | [Q] |
| ORF1015 | A1C_05885 | RBE_0367 | A1I_05935 | A1E_01130 | RC1107 | RF_0181 | RMA_1144 | RP725 | A1G_06140 | ZP_2762 | RT0712 | [C] |
| ORF1017 | A1C_05875 | RBE_0365 | A1I_05950 | A1E_01120 | RC1109 | RF_0179 | RMA_1146 | RP727 | A1G_06150 | ZP_2760 | RT0714 | [S] |
| ORF1018 | A1C_05870 | RBE_0364 | A1I_05955 | A1E_01115 | RC1110 | RF_0178 | RMA_1147 | RP730 | A1G_06155 | ZP_2759 | RT0716 | [S] |
| ORF1019 | A1C_05865 | RBE_0363 | A1I_05960 | A1E_01110 | RC1111 | RF_0177 | RMA_1148 | RP731 | A1G_06160 | ZP_2758 | RT0717 | [H] |
| ORF1020 | A1C_05860 | RBE_0362 | A1I_05965 | A1E_01105 | RC1112 | RF_0176 | RMA_1149 | RP732 | A1G_06165 | ZP_2757 | RT0718 | [L] |
| ORF1022 | A1C_05850 | RBE_0358 | A1I_05990 | A1E_01095 | RC1114 | RF_0174 | RMA_1151 | RP734 | A1G_06175 | ZP_2755 | RT0719 | [L] |
| ORF1024 | A1C_05845 | RBE_0671 | A1I_04145 | A1E_01175 | RC1116 | RF_0171 | RMA_1152 | RP735 | A1G_06190 | ZP_2753 | RT0720 | [I] |
| ORF1035 | A1C_05815 | RBE_0140 | A1I_07210 | A1E_04750 | RC1136 | RF_0162 | RMA_1165 | RP738 | A1G_06260 | ZP_2743 | RT0723 | [I] |
| ORF1037 | A1C_05810 | RBE_0141 | A1I_07205 | A1E_04755 | RC1138 | RF_0161 | RMA_1166 | RP739 | A1G_06265 | ZP_2742 | RT0724 | [C] |
| ORF1039 | A1C_05795 | RBE_1311 | A1I_00305 | A1E_04760 | RC1141 | RF_1185 | RMA_1168 | RP740 | A1G_06280 | ZP_2739 | RT0725 | [R] |
| ORF1040 | A1C_05790 | RBE_1310 | A1I_00310 | A1E_04765 | RC1142 | RF_1186 | RMA_1169 | RP741 | A1G_06285 | ZP_2738 | RT0726 | [R] |
| ORF1043 | A1C_05775 | RBE_1309 | A1I_00315 | A1E_04770 | RC1145 | RF_1187 | RMA_1171 | RP742 | A1G_06300 | ZP_2735 | RT0727 | [H] |
| ORF1044 | A1C_05770 | RBE_1308 | A1I_00320 | A1E_04775 | RC1146 | RF_1188 | RMA_1172 | RP743 | A1G_06305 | ZP_2734 | RT0728 | [E] |
| ORF1045 | A1C_05745 | RBE_1307 | A1I_00325 | A1E_04780 | RC1147 | RF_1189 | RMA_1173 | RP744 | A1G_06310 | ZP_2733 | RT0729 | [R] |
| ORF1047 | A1C_05735 | RBE_1306 | A1I_00335 | A1E_04790 | RC1149 | RF_1191 | RMA_1175 | RP746 | A1G_06320 | ZP_2731 | RT0731 | [L] |
| ORF1053 | A1C_05715 | RBE_1413 | A1I_07865 | A1E_04795 | RC1158 | RF_1194 | RMA_1182 | RP747 | A1G_06370 | ZP_2723 | RT0732 | [R] |
| ORF1054 | A1C_05710 | RBE_1412 | A1I_07860 | A1E_04800 | RC1159 | RF_1195 | RMA_1183 | RP748 | A1G_06375 | ZP_2722 | RT0733 | No hit |
| ORF1055 | A1C_05705 | RBE_1410 | A1I_07850 | A1E_04805 | RC1160 | RF_1196 | RMA_1184 | RP749 | A1G_06380 | ZP_2721 | RT0734 | [U] |
| ORF1056 | A1C_05700 | RBE_1409 | A1I_07845 | A1E_04810 | RC1161 | RF_1197 | RMA_1185 | RP750 | A1G_06385 | ZP_2720 | RT0735 | [I] |
| ORF1057 | A1C_05695 | RBE_1406 | A1I_07830 | A1E_04815 | RC1162 | RF_1198 | RMA_1186 | RP751 | A1G_06390 | ZP_2719 | RT0736 | [J] |
| ORF1058 | A1C_05690 | RBE_1405 | A1I_07825 | A1E_04820 | RC1163 | RF_1199 | RMA_1187 | RP752 | A1G_06395 | ZP_2718 | RT0737 | [J] |
| ORF1059 | A1C_05685 | RBE_1404 | A1I_07820 | A1E_04825 | RC1164 | RF_1200 | RMA_1188 | RP753 | A1G_06400 | ZP_2717 | RT0738 | [E] |
| ORF1060 | A1C_05680 | RBE_1403 | A1I_07815 | A1E_04830 | RC1165 | RF_1201 | RMA_1189 | RP754 | A1G_06405 | ZP_2716 | RT0739 | No hit |
| ORF1065 | A1C_05655 | RBE_0042 | A1I_00690 | A1E_04835 | RC1170 | RF_1208 | RMA_1192 | RP755 | A1G_06435 | ZP_2711 | RT0740 | [GEPR] |
| ORF1070 | A1C_05635 | RBE_0038 | A1I_00710 | A1E_04850 | RC1174 | RF_1213 | RMA_1197 | RP758 | A1G_06460 | ZP_2707 | RT0744 | [E] |
| ORF1071 | A1C_05630 | RBE_0037 | A1I_00195 | A1E_04855 | RC1175 | RF_1214 | RMA_1198 | RP759 | A1G_06465 | ZP_2706 | RT0745 | [R] |
| ORF1072 | A1C_05625 | RBE_1196 | A1I_01300 | A1E_04860 | RC1176 | RF_1215 | RMA_1199 | RP760 | A1G_06470 | ZP_2705 | RT0746 | No hit |
| ORF1077 | A1C_05930 | RBE_0117 | A1I_07345 | A1E_04865 | RC1182 | RF_1221 | RMA_1203 | RP761 | A1G_06490 | ZP_2701 | RT0747 | [L] |
| ORF1078 | A1C_05935 | RBE_0116 | A1I_07350 | A1E_04870 | RC1183 | RF_1222 | RMA_1204 | RP762 | A1G_06495 | ZP_2700 | RT0748 | [IQR] |
| ORF1079 | A1C_05940 | RBE_0115 | A1I_07355 | A1E_04875 | RC1185 | RF_1223 | RMA_1205 | RP763 | A1G_06505 | ZP_2699 | RT0749 | [IQ] |
| ORF1080 | A1C_05945 | RBE_0114 | A1I_07360 | A1E_04880 | RC1186 | RF_1224 | RMA_1206 | RP764 | A1G_06510 | ZP_2698 | RT0750 | [IQ] |
| ORF1086 | A1C_05985 | RBE_0134 | A1I_07245 | A1E_04920 | RC1194 | RF_1229 | RMA_1210 | RP765 | A1G_06535 | ZP_2691 | RT0752 | [F] |
| ORF1087 | A1C_05990 | RBE_0133 | A1I_07250 | A1E_04925 | RC1195 | RF_1230 | RMA_1211 | RP766 | A1G_06540 | ZP_2690 | RT0753 | [K] |
| ORF1088 | A1C_05995 | RBE_0132 | A1I_07255 | A1E_04930 | RC1197 | RF_1231 | RMA_1212 | RP767 | A1G_06545 | ZP_2689 | RT0754 | [M] |
| ORF1089 | A1C_06000 | RBE_0131 | A1I_07265 | A1E_04935 | RC1198 | RF_1232 | RMA_1213 | RP768 | A1G_06550 | ZP_2688 | RT0755 | [D] |
| ORF1090 | A1C_06005 | RBE_0130 | A1I_07270 | A1E_04940 | RC1199 | RF_1233 | RMA_1214 | RP769 | A1G_06555 | ZP_2687 | RT0756 | [R] |
| ORF1091 | A1C_06010 | RBE_0129 | A1I_07275 | A1E_04945 | RC1200 | RF_1234 | RMA_1215 | RP771 | A1G_06560 | ZP_2686 | RT0758 | [M] |
| ORF1093 | A1C_06015 | RBE_0128 | A1I_07280 | A1E_04950 | RC1202 | RF_1235 | RMA_1216 | RP772 | A1G_06570 | ZP_2684 | RT0759 | [I] |
| ORF1094 | A1C_06020 | RBE_0127 | A1I_07285 | A1E_04955 | RC1203 | RF_1236 | RMA_1217 | RP773 | A1G_06575 | ZP_2683 | RT0760 | [J] |
| ORF1095 | A1C_06025 | RBE_0126 | A1I_07290 | A1E_04960 | RC1204 | RF_1237 | RMA_1218 | RP774 | A1G_06580 | ZP_2682 | RT0761 | [J] |
| ORF1096 | A1C_06030 | RBE_0028 | A1I_00145 | A1E_04965 | RC1205 | RF_1238 | RMA_1219 | RP775 | A1G_06585 | ZP_2681 | RT0762 | [U] |
| ORF1097 | A1C_06035 | RBE_1207 | A1I_01235 | A1E_04970 | RC1206 | RF_1239 | RMA_1220 | RP776 | A1G_06590 | ZP_2680 | RT0763 | [L] |
| ORF1103 | A1C_06050 | RBE_0074 | A1I_07575 | A1E_04975 | RC1211 | RF_1244 | RMA_1228 | RP778 | A1G_06640 | ZP_2676 | RT0765 | [L] |
| ORF1104 | A1C_06055 | RBE_0075 | A1I_07570 | A1E_04980 | RC1212 | RF_1245 | RMA_1229 | RP779 | A1G_06645 | ZP_2675 | RT0766 | [M] |
| ORF1105 | A1C_06060 | RBE_0076 | A1I_07565 | A1E_04985 | RC1213 | RF_1246 | RMA_1230 | RP780 | A1G_06650 | ZP_2674 | RT0767 | No hit |
| ORF1106 | A1C_06065 | RBE_0077 | A1I_07560 | A1E_04990 | RC1214 | RF_1247 | RMA_1231 | RP781 | A1G_06655 | ZP_2673 | RT0768 | [GEPR] |
| ORF1107 | A1C_06070 | RBE_0078 | A1I_07555 | A1E_04995 | RC1215 | RF_1248 | RMA_1232 | RP782 | A1G_06660 | ZP_2672 | RT0769 | [U] |
| ORF1108 | A1C_06075 | RBE_0079 | A1I_07550 | A1E_05000 | RC1216 | RF_1249 | RMA_1233 | RP783 | A1G_06665 | ZP_2671 | RT0770 | [J] |
| ORF1109 | A1C_06080 | RBE_0080 | A1I_07545 | A1E_05005 | RC1217 | RF_1250 | RMA_1234 | RP784 | A1G_06670 | ZP_2670 | RT0771 | [U] |
| ORF1110 | A1C_06085 | RBE_0081 | A1I_07540 | A1E_05010 | RC1218 | RF_1251 | RMA_1235 | RP785 | A1G_06675 | ZP_2669 | RT0773 | No hit |
| ORF1111 | A1C_06090 | RBE_0082 | A1I_07535 | A1E_05015 | RC1219 | RF_1252 | RMA_1236 | RP786 | A1G_06680 | ZP_2668 | RT0774 | [P] |
| ORF1112 | A1C_06095 | RBE_0083 | A1I_07530 | A1E_05020 | RC1220 | RF_1253 | RMA_1237 | RP788 | A1G_06685 | ZP_2667 | RT0775 | [M] |
| ORF1113 | A1C_06100 | RBE_0084 | A1I_07525 | A1E_05030 | RC1221 | RF_1254 | RMA_1238 | RP789 | A1G_06690 | ZP_2666 | RT0776 | [QR] |
| ORF1115 | A1C_06105 | RBE_0085 | A1I_07520 | A1E_05035 | RC1224 | RF_1255 | RMA_1241 | RP790 | A1G_06705 | ZP_2663 | RT0777 | [C] |
| ORF1116 | A1C_06110 | RBE_0086 | A1I_07515 | A1E_05040 | RC1225 | RF_1256 | RMA_1242 | RP791 | A1G_06710 | ZP_2662 | RT0778 | [C] |
| ORF1117 | A1C_06115 | RBE_0087 | A1I_07510 | A1E_05045 | RC1226 | RF_1257 | RMA_1243 | RP792 | A1G_06715 | ZP_2661 | RT0779 | [CP] |
| ORF1118 | A1C_06120 | RBE_0088 | A1I_07505 | A1E_05050 | RC1227 | RF_1258 | RMA_1244 | RP793 | A1G_06720 | ZP_2660 | RT0780 | [C] |
| ORF1119 | A1C_06130 | RBE_0089 | A1I_07490 | A1E_05055 | RC1228 | RF_1259 | RMA_1245 | RP794 | A1G_06725 | ZP_2659 | RT0781 | [O] |
| ORF1120 | A1C_06135 | RBE_0103 | A1I_07420 | A1E_05070 | RC1229 | RF_1260 | RMA_1246 | RP795 | A1G_06730 | ZP_2658 | RT0782 | [C] |
| ORF1121 | A1C_06145 | RBE_0102 | A1I_07425 | A1E_05080 | RC1230 | RF_1261 | RMA_1247 | RP796 | A1G_06735 | ZP_2657 | RT0783 | [C] |
| ORF1122 | A1C_06150 | RBE_0101 | A1I_07430 | A1E_05085 | RC1231 | RF_1262 | RMA_1248 | RP797 | A1G_06740 | ZP_2656 | RT0784 | [C] |
| ORF1123 | A1C_06155 | RBE_0100 | A1I_07435 | A1E_05090 | RC1232 | RF_1263 | RMA_1249 | RP798 | A1G_06745 | ZP_2655 | RT0785 | [S] |
| ORF1124 | A1C_06180 | RBE_0098 | A1I_07445 | A1E_05095 | RC1233 | RF_1264 | RMA_1250 | RP799 | A1G_06750 | ZP_2654 | RT0786 | [C] |
| ORF1125 | A1C_06185 | RBE_0097 | A1I_07455 | A1E_05100 | RC1234 | RF_1265 | RMA_1251 | RP800 | A1G_06755 | ZP_2653 | RT0787 | [C] |
| ORF1126 | A1C_06190 | RBE_0096 | A1I_07460 | A1E_05105 | RC1235 | RF_1266 | RMA_1252 | RP801 | A1G_06760 | ZP_2652 | RT0788 | [C] |
| ORF1127 | A1C_06195 | RBE_0095 | A1I_07465 | A1E_05110 | RC1236 | RF_1267 | RMA_1253 | RP802 | A1G_06765 | ZP_2651 | RT0789 | [C] |
| ORF1128 | A1C_06200 | RBE_0094 | A1I_07470 | A1E_05115 | RC1237 | RF_1268 | RMA_1254 | RP803 | A1G_06770 | ZP_2650 | RT0790 | [C] |
| ORF1129 | A1C_06205 | RBE_0092 | A1I_07475 | A1E_05120 | RC1238 | RF_1269 | RMA_1255 | RP804 | A1G_06775 | ZP_2649 | RT0791 | [C] |
| ORF1130 | A1C_06210 | RBE_0091 | A1I_07480 | A1E_05125 | RC1239 | RF_1270 | RMA_1256 | RP805 | A1G_06780 | ZP_2648 | RT0792 | [C] |
| ORF1136 | A1C_06240 | RBE_0108 | A1I_07390 | A1E_05135 | RC1245 | RF_1276 | RMA_1261 | RP807 | A1G_06815 | ZP_2642 | RT0794 | [M] |
| ORF1137 | A1C_06245 | RBE_0111 | A1I_07380 | A1E_05140 | RC1246 | RF_1278 | RMA_1262 | RP808 | A1G_06820 | ZP_2641 | RT0795 | [J] |
| ORF1141 | A1C_06270 | RBE_0016 | A1I_00075 | A1E_05175 | RC1251 | RF_1282 | RMA_1266 | RP809 | A1G_06865 | ZP_2637 | RT0797 | [S] |
| ORF1142 | A1C_06275 | RBE_0017 | A1I_00080 | A1E_05180 | RC1252 | RF_1283 | RMA_1267 | RP810 | A1G_06870 | ZP_2636 | RT0798 | [P] |
| ORF1143 | A1C_06280 | RBE_0018 | A1I_00085 | A1E_05185 | RC1253 | RF_1284 | RMA_1268 | RP811 | A1G_06875 | ZP_2635 | RT0799 | [S] |
| ORF1144 | A1C_06285 | RBE_0019 | A1I_00090 | A1E_05190 | RC1254 | RF_1285 | RMA_1269 | RP812 | A1G_06880 | ZP_2634 | RT0800 | [T] |
| ORF1156 | A1C_06300 | RBE_0144 | A1I_07190 | A1E_05235 | RC1265 | RF_1292 | RMA_1283 | RP814 | A1G_06945 | ZP_2620 | RT0802 | [J] |
| ORF1157 | A1C_06305 | RBE_0143 | A1I_07195 | A1E_05240 | RC1266 | RF_1293 | RMA_1284 | RP815 | A1G_06950 | ZP_2619 | RT0803 | [D] |
| ORF1158 | A1C_06310 | RBE_0178 | A1I_06980 | A1E_05245 | RC1267 | RF_1294 | RMA_1285 | RP816 | A1G_06955 | ZP_2618 | RT0804 | [T] |
| ORF1159 | A1C_06315 | RBE_0177 | A1I_06985 | A1E_05250 | RC1268 | RF_1295 | RMA_1286 | RP817 | A1G_06960 | ZP_2617 | RT0805 | [L] |
| ORF1161 | A1C_06325 | RBE_0175 | A1I_06995 | A1E_05255 | RC1270 | RF_1297 | RMA_1289 | RP819 | A1G_06970 | ZP_2615 | RT0807 | [I] |
| ORF1164 | A1C_06335 | RBE_0172 | A1I_07010 | A1E_05260 | RC1271 | RF_1299 | RMA_1294 | RP821 | A1G_06980 | ZP_2614 | RT0809 | [H] |
| ORF1165 | A1C_06340 | RBE_0171 | A1I_07020 | A1E_05265 | RC1272 | RF_1300 | RMA_1295 | RP822 | A1G_06985 | ZP_2613 | RT0810 | [O] |
| ORF1167 | A1C_06390 | RBE_0154 | A1I_07115 | A1E_05275 | RC1274 | RF_1307 | RMA_1298 | RP823 | A1G_07010 | ZP_2611 | RT0811 | [D] |
| ORF1169 | A1C_06395 | RBE_0156 | A1I_07100 | A1E_05290 | RC1276 | RF_1309 | RMA_1301 | RP824 | A1G_07020 | ZP_2610 | RT0812 | [J] |
| ORF1172 | A1C_06410 | RBE_0158 | A1I_07090 | A1E_05300 | RC1279 | RF_1311 | RMA_1305 | RP825 | A1G_07035 | ZP_2607 | RT0813 | [M] |
| ORF1173 | A1C_06415 | RBE_0159 | A1I_07085 | A1E_05305 | RC1280 | RF_1312 | RMA_1306 | RP826 | A1G_07040 | ZP_2606 | RT0814 | No hit |
| ORF1174 | A1C_06420 | RBE_1270 | A1I_01210 | A1E_05315 | RC1281 | RF_1313 | RMA_1307 | RP827 | A1G_07045 | ZP_2605 | RT0815 | [M] |
| ORF1175 | A1C_06425 | RBE_1271 | A1I_01215 | A1E_05320 | RC1282 | RF_1314 | RMA_1308 | RP828 | A1G_07050 | ZP_2604 | RT0816 | [M] |
| ORF1176 | A1C_06430 | RBE_1272 | A1I_01220 | A1E_05325 | RC1283 | RF_1315 | RMA_1309 | RP829 | A1G_07055 | ZP_2603 | RT0817 | [C] |
| ORF1177 | A1C_06435 | RBE_1273 | A1I_01225 | A1E_05330 | RC1284 | RF_1318 | RMA_1310 | RP830 | A1G_07060 | ZP_2602 | RT0818 | [O] |
| ORF1178 | A1C_06440 | RBE_1426 | A1I_07935 | A1E_05335 | RC1285 | RF_1319 | RMA_1311 | RP831 | A1G_07065 | ZP_2601 | RT0819 | [FJ] |
| ORF1179 | A1C_06445 | RBE_1425 | A1I_07930 | A1E_05340 | RC1286 | RF_1320 | RMA_1312 | RP832 | A1G_07070 | ZP_2600 | RT0820 | [P] |
| ORF1180 | A1C_06450 | RBE_1424 | A1I_07925 | A1E_05345 | RC1287 | RF_1321 | RMA_1313 | RP833 | A1G_07075 | ZP_2599 | RT0821 | [M] |
| ORF1182 | A1C_06460 | RBE_1422 | A1I_07915 | A1E_05355 | RC1289 | RF_1323 | RMA_1315 | RP834 | A1G_07085 | ZP_2597 | RT0822 | [P] |
| ORF1186 | A1C_06465 | RBE_0162 | A1I_07070 | A1E_05360 | RC1294 | RF_1324 | RMA_1319 | RP835 | A1G_07115 | ZP_2592 | RT0823 | [L] |
| ORF1187 | A1C_06470 | RBE_0161 | A1I_07075 | A1E_05365 | RC1295 | RF_1325 | RMA_1320 | RP836 | A1G_07120 | ZP_2591 | RT0824 | [L] |
| ORF1188 | A1C_06475 | RBE_0160 | A1I_07080 | A1E_05370 | RC1296 | RF_1326 | RMA_1321 | RP837 | A1G_07125 | ZP_2590 | RT0825 | No hit |
| ORF1191 | A1C_06510 | RBE_0633 | A1I_04385 | A1E_05380 | RC1301 | RF_1332 | RMA_1324 | RP839 | A1G_07140 | ZP_2587 | RT0827 | [S] |
| ORF1192 | A1C_06515 | RBE_0632 | A1I_04390 | A1E_05385 | RC1302 | RF_1333 | RMA_1325 | RP840 | A1G_07145 | ZP_2586 | RT0828 | [O] |
| ORF1193 | A1C_06520 | RBE_0631 | A1I_04395 | A1E_05390 | RC1303 | RF_1334 | RMA_1326 | RP841 | A1G_07150 | ZP_2585 | RT0829 | [H] |
| ORF1194 | A1C_06530 | RBE_1363 | A1I_00735 | A1E_05395 | RC1306 | RF_1336 | RMA_1328 | RP842 | A1G_07160 | ZP_2583 | RT0830 | [O] |
| ORF1195 | A1C_06535 | RBE_1324 | A1I_00230 | A1E_05400 | RC1307 | RF_1337 | RMA_1329 | RP843 | A1G_07165 | ZP_2582 | RT0831 | [R] |
| ORF1196 | A1C_06540 | RBE_1325 | A1I_00225 | A1E_05405 | RC1308 | RF_1338 | RMA_1330 | RP844 | A1G_07170 | ZP_2581 | RT0832 | [C] |
| ORF1197 | A1C_06550 | RBE_1247 | A1I_00935 | A1E_05420 | RC1309 | RF_1340 | RMA_1332 | RP845 | A1G_07180 | ZP_2580 | RT0833 | [L] |
| ORF1198 | A1C_06555 | RBE_1246 | A1I_00930 | A1E_05425 | RC1310 | RF_1341 | RMA_1333 | RP846 | A1G_07185 | ZP_2579 | RT0834 | [J] |
| ORF1201 | A1C_06570 | RBE_1243 | A1I_00915 | A1E_05430 | RC1314 | RF_1344 | RMA_1334 | RP847 | A1G_07200 | ZP_2575 | RT0836 | [J] |
| ORF1202 | A1C_06575 | RBE_1358 | A1I_00760 | A1E_05435 | RC1315 | RF_1345 | RMA_1335 | RP848 | A1G_07205 | ZP_2574 | RT0837 | [J] |
| ORF1203 | A1C_06580 | RBE_1359 | A1I_00755 | A1E_05440 | RC1316 | RF_1346 | RMA_1336 | RP849 | A1G_07210 | ZP_2573 | RT0838 | [J] |
| ORF1204 | A1C_06585 | RBE_1360 | A1I_00750 | A1E_05445 | RC1317 | RF_1347 | RMA_1337 | RP850 | A1G_07215 | ZP_2572 | RT0839 | [J] |
| ORF1207 | A1C_06595 | RBE_0064 | A1I_00570 | A1E_05450 | RC1320 | RF_1348 | RMA_1340 | RP851 | A1G_07230 | ZP_2569 | RT0840 | [S] |
| ORF1211 | A1C_06610 | RBE_0068 | A1I_00545 | A1E_05470 | RC1324 | RF_1350 | RMA_1382 | RP853 | A1G_07245 | ZP_2565 | RT0842 | No hit |
| ORF1212 | A1C_06615 | RBE_0069 | A1I_00540 | A1E_05475 | RC1325 | RF_1351 | RMA_1381 | RP854 | A1G_07250 | ZP_2564 | RT0843 | No hit |
| ORF1213 | A1C_06620 | RBE_0070 | A1I_00535 | A1E_05480 | RC1326 | RF_1352 | RMA_1380 | RP855 | A1G_07255 | ZP_2563 | RT0844 | No hit |
| ORF1214 | A1C_06625 | RBE_0071 | A1I_00515 | A1E_05485 | RC1327 | RF_1353 | RMA_1379 | RP856 | A1G_07260 | ZP_2562 | RT0845 | [J] |
| ORF1215 | A1C_06660 | RBE_0060 | A1I_00590 | A1E_05490 | RC1328 | RF_1356 | RMA_1378 | RP857 | A1G_07265 | ZP_2561 | RT0846 | [J] |
| ORF1216 | A1C_06665 | RBE_0056 | A1I_00620 | A1E_05495 | RC1329 | RF_1357 | RMA_1377 | RP858 | A1G_07270 | ZP_2560 | RT0847 | [K] |
| ORF1217 | A1C_06670 | RBE_0055 | A1I_00625 | A1E_05500 | RC1330 | RF_1358 | RMA_1376 | RP859 | A1G_07275 | ZP_2559 | RT0848 | [L] |
| ORF1218 | A1C_06675 | RBE_0054 | A1I_00630 | A1E_05505 | RC1331 | RF_1359 | RMA_1375 | RP860 | A1G_07280 | ZP_2558 | RT0849 | [I] |
| ORF1219 | A1C_06680 | RBE_0053 | A1I_00635 | A1E_05510 | RC1332 | RF_1360 | RMA_1374 | RP861 | A1G_07285 | ZP_2557 | RT0850 | [K] |
| ORF1220 | A1C_06685 | RBE_0044 | A1I_00680 | A1E_05515 | RC1333 | RF_1361 | RMA_1373 | RP862 | A1G_07290 | ZP_2556 | RT0851 | [C] |
| ORF1222 | A1C_06695 | RBE_0046 | A1I_00670 | A1E_05540 | RC1335 | RF_1363 | RMA_1371 | RP864 | A1G_07300 | ZP_2554 | RT0853 | [M] |
| ORF1224 | A1C_06705 | RBE_0048 | A1I_00660 | A1E_05550 | RC1337 | RF_1365 | RMA_1369 | RP866 | A1G_07310 | ZP_2552 | RT0857 | [S] |
| ORF1225 | A1C_06710 | RBE_1317 | A1I_00275 | A1E_05555 | RC1339 | RF_1366 | RMA_1368 | RP867 | A1G_07330 | ZP_2551 | RT0858 | [R] |
| ORF1227 | A1C_06720 | RBE_1318 | A1I_00270 | A1E_05560 | RC1341 | RF_1370 | RMA_1365 | RP868 | A1G_07360 | ZP_2549 | RT0859 | [E] |
| ORF1228 | A1C_06725 | RBE_1319 | A1I_00265 | A1E_05565 | RC1342 | RF_1371 | RMA_1364 | RP869 | A1G_07365 | ZP_2548 | RT0860 | [R] |
| ORF1229 | A1C_06730 | RBE_1320 | A1I_00260 | A1E_05570 | RC1343 | RF_1372 | RMA_1363 | RP870 | A1G_07370 | ZP_2547 | RT0861 | [I] |
| ORF1232 | A1C_06735 | RBE_1321 | A1I_00255 | A1E_05575 | RC1345 | RF_1374 | RMA_1360 | RP871 | A1G_07385 | ZP_2545 | RT0862 | [R] |
| ORF1233 | A1C_06740 | RBE_1421 | A1I_07905 | A1E_05580 | RC1346 | RF_1375 | RMA_1359 | RP872 | A1G_07390 | ZP_2544 | RT0863 | [L] |
| ORF1234 | A1C_06750 | RBE_1420 | A1I_07900 | A1E_05585 | RC1347 | RF_1376 | RMA_1358 | RP874 | A1G_07400 | ZP_2543 | RT0865 | [E] |
| ORF1242 | A1C_06780 | RBE_0051 | A1I_00645 | A1E_05600 | RC1356 | RF_1382 | RMA_1350 | RP875 | A1G_07445 | ZP_2535 | RT0866 | [S] |
| ORF1243 | A1C_06785 | RBE_0052 | A1I_00640 | A1E_05610 | RC1357 | RF_1383 | RMA_1349 | RP876 | A1G_07450 | ZP_2534 | RT0867 | [H] |
| ORF1245 | A1C_06795 | RBE_1378 | A1I_07655 | A1E_05620 | RC1359 | RF_1385 | RMA_1347 | RP878 | A1G_07460 | ZP_2532 | RT0869 | [J] |
| ORF1246 | A1C_06800 | RBE_1379 | A1I_07660 | A1E_05625 | RC1360 | RF_1386 | RMA_1346 | RP879 | A1G_07465 | ZP_2531 | RT0870 | [J] |
| ORF1247 | A1C_06805 | RBE_1387 | A1I_07705 | A1E_05630 | RC1361 | RF_1387 | RMA_1345 | RP880 | A1G_07470 | ZP_2530 | RT0871 | [L] |
| ORF1253 | A1C_06855 | RBE_1381 | A1I_07670 | A1E_05655 | RC1368 | RF_1395 | RMA_1384 | RP881 | A1G_07490 | ZP_2525 | RT0873 | [GEPR] |
| ORF1254 | A1C_06860 | RBE_1380 | A1I_07665 | A1E_05660 | RC1369 | RF_1396 | RMA_1385 | RP882 | A1G_07495 | ZP_2524 | RT0874 | [H] |
| ORF1257 | A1C_06870 | RBE_1427 | A1I_07940 | A1E_05665 | RC1372 | RF_1398 | RMA_1387 | RP883 | A1G_07510 | ZP_2522 | RT0875 | [S] |
| ORF1258 | A1C_06875 | RBE_1428 | A1I_07945 | A1E_05670 | RC1373 | RF_1399 | RMA_1388 | RP884 | A1G_07515 | ZP_2521 | RT0876 | [H] |
| ORF1259 | A1C_06880 | RBE_1429 | A1I_07950 | A1E_05675 | RC1374 | RF_1400 | RMA_1389 | RP885 | A1G_07520 | ZP_2520 | RT0877 | [H] |

Note: The core genes are identified using the OrthoMCL program with default parameters and only the set with exact one representative in each genome is considered. The genes are designated by locus tag accession numbers. If the members of an orthologous group can not be assigned to any COG functional category, they will be labeled with “No hit”. The abbreviation for each genome is as follows: raf, *R. africae* ESF-5; rsi, *R. sibirica* 246; rco, *R. conorii* Malish7; rri, *R. rickettsii*; rms, *R. massiliae* MTU5; rak, *R. akari* Hartford; rfe, *R. felis* URRWXCal2; rpr, *R. prowazekii* Madrid E; rty, *R. typhi* Wilmington; rcm, *R. canadensis* McKie; rbo, *R. bellii* OSU 85-389 and *rbe, R. bellii* RML369-C. 7 The abbreviation for each functional category is as follows: [J], Translation, ribosomal structure and biogenesis; [K], transcription; [L], replication, recombination and repair; [D], cell cycle control, cell division, chromosome partitioning; [V], defense mechanisms; [T], signal transduction mechanisms; [M], cell wall/membrane/envelope biogenesis; [N], cell motility; [U], intracellular trafficking, secretion, and vesicular transport; [O], posttranslational modification, protein turnover, chaperones; [C], energy production and conversion; [G], carbohydrate transport and metabolism; [E], amino acid transport and metabolism; [F], nucleotide transport and metabolism; [H], coenzyme transport and metabolism; [I], lipid transport and metabolism; [P], inorganic ion transport and metabolism; [Q], secondary metabolites biosynthesis, transport and catabolism; [R], general function prediction only and [S], function unknown.
