# Supplementary material for: Evidence of Extensive Homologous Recombination in the Core Genome of Rickettsia
Source: Comp Funct Genomics. 2009 May 25;2009:510270. doi: 10.1155/2009/510270 (PMC2685993; doi:10.1155/2009/510270)
Supplement: Supplementary file 2 [file 510270.f2.doc]

**Table S2. *Rickettsia*’s** core genes show significant evidence of recombination.

| **Locus tag** | **Function** | **COG category** | **Detection method** |
| --- | --- | --- | --- |
| A1G_00025 | NADPH-dependent glutamate synthase beta chain | [ER] | RDP, GC, MC, Ch |
| A1G_00100 | ABC-type metal ion transport system, periplasmic component | [P] | RDP, GC, MC, Ch |
| A1G_00105 | tRNA nucleotidyltransferase/poly(A) polymerase | [J] | RDP, GC, MC, Ch |
| A1G_00130 | No hits | No hit | RDP, GC, MC, Ch |
| A1G_00165 | F0F1-type ATP synthase, subunit b | [C] | GC, MC, Ch |
| A1G_00300 | No hits | No hit | RDP, MC, Ch |
| A1G_00380 | ATPases with chaperone activity, ATP-binding subunit | [O] | RDP, GC, MC, Ch |
| A1G_00420 | Predicted ATPase of the PP-loop superfamily implicated in cell cycle control | [D] | RDP, MC, Ch |
| A1G_00475 | Preprotein translocase subunit YidC | [U] | RDP, GC, MC, Ch |
| A1G_00585 | Arginyl-tRNA synthetase | [J] | RDP, GC, MC, Ch |
| A1G_00595 | Type IIA topoisomerase (DNA gyrase/topo II, topoisomerase IV), A subunit | [L] | MC, Ch |
| A1G_00650 | Permeases of the major facilitator superfamily | [GEPR] | MC, Ch |
| A1G_00675 | Cysteinyl-tRNA synthetase | [J] | RDP, Ch |
| A1G_00750 | Alanine racemase | [M] | MC, Ch |
| A1G_00800 | Predicted GTPase | [R] | RDP, Ch |
| A1G_00815 | Type IV secretory pathway, VirB4 components | [U] | MC, Ch |
| A1G_00820 | Type IV secretory pathway, VirB6 components | [U] | RDP, GC, MC, Ch |
| A1G_00825 | Type IV secretory pathway, VirB6 components | [U] | RDP, GC, MC |
| A1G_00830 | Type IV secretory pathway, VirB6 components | [U] | RDP, GC, MC, Ch |
| A1G_00875 | tRNA-(guanine-N1)-methyltransferase | [J] | RDP, MC, Ch |
| A1G_00900 | Signal peptidase I | [U] | RDP, GC, MC |
| A1G_00935 | Predicted nucleoside-diphosphate-sugar epimerase | [M] | RDP, GC, MC, Ch |
| A1G_00940 | ATPases involved in chromosome partitioning | [D] | MC, Ch |
| A1G_00955 | Trypsin-like serine proteases, typically periplasmic | [O] | RDP, MC, Ch |
| A1G_00985 | ABC-type amino acid transport system, permease component | [E] | MC, Ch |
| A1G_01055 | ATPases involved in chromosome partitioning | [D] | MC, Ch |
| A1G_01065 | Aspartyl-tRNA synthetase | [J] | RDP, MC, Ch |
| A1G_01100 | Asp-tRNAAsn/Glu-tRNAGln amidotransferase A subunit | [J] | RDP, MC, Ch |
| A1G_01150 | Outer membrane protein/protective antigen OMA87 | [M] | RDP, Ch |
| A1G_01210 | ATPase involved in DNA replication | [L] | MC, Ch |
| A1G_01300 | 2-oxoglutarate dehydrogenase complex and related enzymes | [C] | RDP, GC, MC, Ch |
| A1G_01315 | DNA uptake lipoprotein | [R] | MC, Ch |
| A1G_01330 | DnaJ-class molecular chaperone with C-terminal Zn finger domain | [O] | MC, Ch |
| A1G_01335 | Molecular chaperone | [O] | RDP, Ch |
| A1G_01360 | DNA polymerase III, delta subunit | [L] | GC, Ch |
| A1G_01365 | Ubiquinone biosynthesis protein COQ7 | [H] | MC, Ch |
| A1G_01490 | No hits | No hit | RDP, GC |
| A1G_01525 | Glutaredoxin and related proteins | [O] | MC, Ch |
| A1G_01555 | Type IIA topoisomerase (DNA gyrase/topo II, topoisomerase IV), A subunit | [L] | RDP, GC, MC, Ch |
| A1G_01595 | N-formylmethionyl-tRNA deformylase | [J] | MC, Ch |
| A1G_01600 | Methionyl-tRNA formyltransferase | [J] | RDP, GC |
| A1G_01615 | Predicted ATPase | [R] | MC, Ch |
| A1G_01640 | ABC-type multidrug transport system, ATPase and permease components | [V] | MC, Ch |
| A1G_01670 | Glycosyltransferases involved in cell wall biogenesis | [M] | RDP, MC, Ch |
| A1G_01675 | Predicted Zn-dependent peptidases | [R] | RDP, GC, MC |
| A1G_01690 | Threonyl-tRNA synthetase | [J] | RDP, GC, MC, Ch |
| A1G_01745 | Outer membrane protein | [MU] | GC, MC |
| A1G_01760 | FOG: Ankyrin repeat | [R] | RDP, Ch |
| A1G_01800 | Ribosomal protein S9 | [J] | RDP, GC, MC, Ch |
| A1G_01900 | UDP-N-acetylmuramate-alanine ligase | [M] | RDP, GC, MC, Ch |
| A1G_01905 | UDP-N-acetylmuramate dehydrogenase | [M] | MC, Ch |
| A1G_01910 | D-alanine-D-alanine ligase and related ATP-grasp enzymes | [M] | MC, Ch |
| A1G_01940 | UDP-3-O-acyl-N-acetylglucosamine deacetylase | [M] | MC, Ch |
| A1G_01985 | Pyruvate/2-oxoglutarate dehydrogenase complex, dehydrogenase component | [C] | RDP, MC, Ch |
| A1G_01995 | Predicted membrane GTPase involved in stress response | [T] | RDP, Ch |
| A1G_02025 | Multisubunit Na+/H+ antiporter, MnhG subunit | [P] | GC, MC, Ch |
| A1G_02030 | Multisubunit Na+/H+ antiporter, MnhB subunit | [P] | RDP, GC, MC |
| A1G_02035 | No hits | No hit | RDP, GC |
| A1G_02040 | No hits | No hit | RDP, GC, MC, Ch |
| A1G_02100 | Membrane GTPase LepA | [M] | GC, MC, Ch |
| A1G_02165 | Protease II | [E] | GC, MC, Ch |
| A1G_02190 | Formate hydrogenlyase subunit 3/Multisubunit Na+/H+ antiporter | [CP] | RDP, GC, MC, Ch |
| A1G_02230 | Type IV secretory pathway, VirB10 components | [U] | RDP, MC |
| A1G_02240 | Type IV secretory pathway, VirD4 components | [U] | MC, Ch |
| A1G_02275 | No hits | No hit | RDP, MC, Ch |
| A1G_02285 | 3'-Phosphoadenosine 5'-phosphosulfate (PAPS) 3'-phosphatase | [P] | MC, Ch |
| A1G_02440 | Aspartate-semialdehyde dehydrogenase | [E] | RDP, MC, Ch |
| A1G_02490 | Lipid A disaccharide synthetase | [M] | RDP, GC, MC, Ch |
| A1G_02560 | Membrane protease subunits, stomatin/prohibitin homologs | [O] | GC, MC, Ch |
| A1G_02570 | Predicted membrane-associated, metal-dependent hydrolase | [R] | RDP, GC, MC, Ch |
| A1G_02590 | dTDP-4-dehydrorhamnose reductase | [M] | RDP, MC, Ch |
| A1G_02595 | Predicted nucleoside-diphosphate sugar epimerases | [MG] | GC, Ch |
| A1G_02600 | UDP-N-acetylglucosamine 2-epimerase | [M] | MC, Ch |
| A1G_02625 | No hits | No hit | RDP, GC, MC, Ch |
| A1G_02715 | 3-methyladenine DNA glycosylase | [L] | RDP, MC, Ch |
| A1G_02760 | Lipid A core - O-antigen ligase and related enzymes | [M] | RDP, GC, MC, Ch |
| A1G_02790 | No hits | No hit | RDP, GC, MC, Ch |
| A1G_02875 | Malic enzyme | [C] | RDP, GC |
| A1G_02900 | Permeases of the major facilitator superfamily | [GEPR] | RDP, GC |
| A1G_02950 | Malate/lactate dehydrogenases | [C] | RDP, MC, Ch |
| A1G_02990 | GTP cyclohydrolase I | [H] | RDP, MC |
| A1G_03030 | ABC-type multidrug transport system, ATPase and permease components | [V] | RDP, GC, MC, Ch |
| A1G_03045 | Lipoproteins | [M] | RDP, GC, MC, Ch |
| A1G_03050 | Predicted periplasmic or secreted lipoprotein | [R] | RDP, GC, MC, Ch |
| A1G_03060 | Intracellular septation protein A | [D] | RDP, Ch |
| A1G_03075 | No hits | No hit | RDP, GC |
| A1G_03080 | Protocatechuate 3,4-dioxygenase beta subunit | [Q] | MC, Ch |
| A1G_03085 | Thiol-disulfide isomerase and thioredoxins | [OC] | RDP, GC, MC, Ch |
| A1G_03100 | Soluble lytic murein transglycosylase and related regulatory proteins | [M] | RDP, GC, MC, Ch |
| A1G_03115 | RecB family exonuclease | [L] | RDP, GC, MC, Ch |
| A1G_03145 | Membrane proteins related to metalloendopeptidases | [M] | RDP, MC |
| A1G_03160 | UDP-N-acetylmuramoylalanine-D-glutamate ligase | [M] | RDP, MC, Ch |
| A1G_03170 | UDP-N-acetylglucosamine:LPS N-acetylglucosamine transferase | [M] | RDP, MC, Ch |
| A1G_03250 | Glycosyltransferase | [M] | RDP, GC, MC, Ch |
| A1G_03275 | 2-methylthioadenine synthetase | [J] | MC, Ch |
| A1G_03285 | Phenylalanyl-tRNA synthetase beta subunit | [J] | RDP, MC, Ch |
| A1G_03340 | Branched-chain amino acid aminotransferase | [EH] | MC, Ch |
| A1G_03365 | Succinyl-CoA synthetase, beta subunit | [C] | RDP, MC, Ch |
| A1G_03605 | Thioredoxin reductase | [O] | RDP, GC |
| A1G_03725 | tRNA delta(2)-isopentenylpyrophosphate transferase | [J] | GC, MC, Ch |
| A1G_03735 | Phosphomannomutase | [G] | RDP, GC, MC, Ch |
| A1G_03770 | Polyribonucleotide nucleotidyltransferase (polynucleotide phosphorylase) | [J] | GC, MC |
| A1G_03790 | ATPase involved in DNA repair | [L] | RDP, GC, MC, Ch |
| A1G_03865 | Folylpolyglutamate synthase | [H] | RDP, GC |
| A1G_03895 | Biotin-(acetyl-CoA carboxylase) ligase | [H] | RDP, GC, MC, Ch |
| A1G_03940 | Protein chain release factor A | [J] | MC, Ch |
| A1G_03945 | Single-stranded DNA-specific exonuclease | [L] | RDP, GC, MC, Ch |
| A1G_03955 | Transcription termination factor | [K] | MC, Ch |
| A1G_04030 | Protein required for attachment to host cells | [N] | MC, Ch |
| A1G_04070 | Permeases of the major facilitator superfamily | [GEPR] | GC, MC, Ch |
| A1G_04100 | Geranylgeranyl pyrophosphate synthase | [H] | RDP, Ch |
| A1G_04105 | Xaa-Pro aminopeptidase | [E] | RDP, GC, MC, Ch |
| A1G_04130 | Cysteine sulfinate desulfinase/cysteine desulfurase and related enzymes | [E] | RDP, GC, MC, Ch |
| A1G_04195 | L-lactate dehydrogenase and related alpha-hydroxy acid dehydrogenases | [C] | MC, Ch |
| A1G_04215 | Cytidylate kinase | [F] | MC, Ch |
| A1G_04315 | Predicted aminomethyltransferase related to GcvT | [R] | MC, Ch |
| A1G_04345 | SAM-dependent methyltransferases | [QR] | RDP, GC, MC, Ch |
| A1G_04370 | No hits | No hit | GC, MC, Ch |
| A1G_04400 | No hit conserved protein | [S] | MC, Ch |
| A1G_04480 | NADH:ubiquinone oxidoreductase subunit 2 (chain N) | [C] | MC, Ch |
| A1G_04510 | Primosomal protein N' (replication factor Y) - superfamily II helicase | [L] | RDP, GC, MC, Ch |
| A1G_04525 | Replicative DNA helicase | [L] | RDP, GC, MC, Ch |
| A1G_04570 | No hits | No hit | GC, MC, Ch |
| A1G_04595 | Translation initiation factor 2 (IF-2; GTPase) | [J] | RDP, Ch |
| A1G_04720 | No hit low-complexity proteins | [S] | RDP, GC |
| A1G_04725 | No hits | No hit | RDP, MC, Ch |
| A1G_04755 | Predicted S-adenosylmethionine-dependent methyltransferase | [M] | RDP, MC |
| A1G_04780 | Nuclease subunit of the excinuclease complex | [L] | RDP, GC, MC, Ch |
| A1G_04880 | Type IIA topoisomerase (DNA gyrase/topo II, topoisomerase IV), B subunit | [L] | RDP, MC, Ch |
| A1G_04885 | Predicted sugar phosphatases of the HAD superfamily | [G] | MC, Ch |
| A1G_04960 | No hit membrane protein, putative virulence factor | [R] | RDP, MC |
| A1G_04965 | No hits | No hit | RDP, MC, Ch |
| A1G_05030 | UDP-N-acetylmuramyl pentapeptide synthase | [M] | RDP, GC, MC, Ch |
| A1G_05035 | UDP-N-acetylmuramyl tripeptide synthase | [M] | GC, MC, Ch |
| A1G_05040 | Transcription-repair coupling factor (superfamily II helicase) | [LK] | RDP, GC, MC, Ch |
| A1G_05085 | Patatin | [R] | RDP, GC, MC, Ch |
| A1G_05240 | Isoleucyl-tRNA synthetase | [J] | RDP, GC, MC, Ch |
| A1G_05265 | Acetyl/propionyl-CoA carboxylase, alpha subunit | [I] | MC, Ch |
| A1G_05305 | Glutamyl- and glutaminyl-tRNA synthetases | [J] | RDP, GC, MC, Ch |
| A1G_05320 | Chaperonin GroEL (HSP60 family) | [O] | RDP, GC |
| A1G_05650 | Exonuclease VII, large subunit | [L] | GC, MC, Ch |
| A1G_05725 | Predicted unusual protein kinase | [R] | RDP, GC, MC, Ch |
| A1G_05805 | Mg-dependent DNase | [L] | RDP, GC, MC, Ch |
| A1G_05820 | Permeases of the major facilitator superfamily | [GEPR] | RDP, GC, MC, Ch |
| A1G_05850 | Valyl-tRNA synthetase | [J] | RDP, MC, Ch |
| A1G_05870 | No hit protein conserved in bacteria | [S] | RDP, MC |
| A1G_05925 | ATP-dependent protease Clp, ATPase subunit | [O] | MC, Ch |
| A1G_05935 | Predicted PP-loop superfamily ATPase | [R] | MC, Ch |
| A1G_05950 | ABC-type multidrug transport system, ATPase and permease components | [V] | MC, Ch |
| A1G_05975 | No hits | No hit | RDP, GC, MC, Ch |
| A1G_06030 | Large exoproteins involved in heme utilization or adhesion | [U] | RDP, GC, MC, Ch |
| A1G_06040 | Beta-glucosidase-related glycosidases | [G] | RDP, GC, MC, Ch |
| A1G_06065 | Lauroyl/myristoyl acyltransferase | [M] | GC, MC, Ch |
| A1G_06095 | Queuine/archaeosine tRNA-ribosyltransferase | [J] | RDP, GC |
| A1G_06155 | No hit protein conserved in bacteria | [S] | RDP, MC, Ch |
| A1G_06160 | Dephospho-CoA kinase | [H] | RDP, GC |
| A1G_06165 | DNA polymerase III, epsilon subunit and related 3'-5' exonucleases | [L] | MC, Ch |
| A1G_06190 | (acyl-carrier-protein) S-malonyltransferase | [I] | RDP, GC, MC, Ch |
| A1G_06260 | Poly(3-hydroxyalkanoate) synthetase | [I] | MC, Ch |
| A1G_06265 | ATP/ADP translocase | [C] | MC, Ch |
| A1G_06300 | Lipoate synthase | [H] | RDP, GC |
| A1G_06405 | No hits | No hit | RDP, GC, MC, Ch |
| A1G_06460 | NAD-specific glutamate dehydrogenase | [E] | RDP, Ch |
| A1G_06465 | Predicted GTPase | [R] | MC, Ch |
| A1G_06470 | No hits | No hit | RDP, GC, MC, Ch |
| A1G_06490 | RecA/RadA recombinase | [L] | MC, Ch |
| A1G_06590 | DNA polymerase I - 3'-5' exonuclease and polymerase domains | [L] | GC, MC, Ch |
| A1G_06640 | DNA polymerase III, alpha subunit | [L] | RDP, GC, MC, Ch |
| A1G_06655 | Permeases of the major facilitator superfamily | [GEPR] | GC, MC |
| A1G_06675 | No hits | No hit | RDP, GC, MC, Ch |
| A1G_06680 | Membrane protein TerC, possibly involved in tellurium resistance | [P] | RDP, GC |
| A1G_06690 | SAM-dependent methyltransferases | [QR] | RDP, GC |
| A1G_06715 | NADH:ubiquinone oxidoreductase subunit 5/Multisubunit Na+/H+ antiporter | [CP] | RDP, GC, MC, Ch |
| A1G_06740 | NADH dehydrogenase/NADH:ubiquinone oxidoreductase 75 kD subunit | [C] | GC, MC, Ch |
| A1G_06750 | Aconitase A | [C] | RDP, GC, MC, Ch |
| A1G_06980 | 3-polyprenyl-4-hydroxybenzoate decarboxylase and related decarboxylases | [H] | RDP, GC, MC |
| A1G_07010 | DNA segregation ATPase FtsK/SpoIIIE and related proteins | [D] | RDP, MC |
| A1G_07045 | Opacity protein and related surface antigens | [M] | MC, Ch |
| A1G_07055 | Ferredoxin | [C] | GC, MC, Ch |
| A1G_07060 | ABC-type transport system (cytochrome c biogenesis), permease component | [O] | MC, Ch |
| A1G_07125 | No hits | No hit | MC, Ch |
| A1G_07170 | Citrate synthase | [C] | RDP, MC, Ch |
| A1G_07180 | Uracil-DNA glycosylase | [L] | MC, Ch |
| A1G_07185 | Pseudouridylate synthases, 23S RNA-specific | [J] | RDP, GC |
| A1G_07200 | Methylase of polypeptide chain release factors | [J] | RDP, GC, MC, Ch |
| A1G_07205 | Putative translation factor (SUA5) | [J] | MC, Ch |
| A1G_07210 | Glycyl-tRNA synthetase, beta subunit | [J] | MC, Ch |
| A1G_07260 | Alanyl-tRNA synthetase | [J] | RDP, GC, MC, Ch |
| A1G_07275 | DNA primase (bacterial type) | [L] | RDP, GC, MC, Ch |
| A1G_07300 | Outer membrane lipoprotein-sorting protein | [M] | MC, Ch |
| A1G_07365 | Metal-dependent hydrolases of the beta-lactamase superfamily I | [R] | GC, MC, Ch |
| A1G_07400 | Acetylornithine deacetylase/Succinyl-diaminopimelate desuccinylase | [E] | RDP, MC, Ch |
| A1G_07445 | No hit conserved protein | [S] | MC, Ch |
| A1G_07520 | Uroporphyrinogen-III decarboxylase | [H] | MC, Ch |

Note: only recombinations that are supported by at least two independent recombination detection methods are shown. The abbreviation for each recombination method is as follow: RDP, RDP; GC, GENECONV; MC, MaxChi and Ch, Chimaera.
